# Molecular Recognition of Parallel G-quadruplex [d-(TTGGGGT)]_4_ Containing *Tetrahymena* Telomeric DNA Sequence by Anticancer Drug Daunomycin: NMR-Based Structure and Thermal Stability

**DOI:** 10.3390/molecules23092266

**Published:** 2018-09-05

**Authors:** Ritu Barthwal, Zia Tariq

**Affiliations:** Department of Biotechnology, Indian Institute of Technology Roorkee, Roorkee 247667, India; ziatariq88@gmail.com

**Keywords:** daunomycin, G-quadruplex DNA, surface plasmon resonance, circular dichroism, nuclear magnetic resonance, intermolecular NOEs, restrained molecular dynamics simulations, differential scanning calorimetry, thermal stabilization

## Abstract

The anticancer drug daunomycin exerts its influence by multiple strategies of action to interfere with gene functioning. Besides inhibiting DNA/RNA synthesis and topoisomerase-II, it affects the functional pathway of telomere maintenance by the telomerase enzyme. We present evidence of the binding of daunomycin to parallel-stranded tetramolecular [d-(TTGGGGT)]_4_ guanine (G)-quadruplex DNA comprising telomeric DNA from *Tetrahymena thermophilia* by surface plasmon resonance and Diffusion Ordered SpectroscopY (DOSY). Circular Dichroism (CD) spectra show the disruption of daunomycin dimers, suggesting the end-stacking and groove-binding of the daunomycin monomer. Proton and phosphorus-31 Nuclear Magnetic Resonance (NMR) spectroscopy show a sequence-specific interaction and a clear proof of absence of intercalation of the daunomycin chromophore between base quartets or stacking between G-quadruplexes. Restrained molecular dynamics simulations using observed short interproton distance contacts depict interaction at the molecular level. The interactions involving ring A and daunosamine protons, the stacking of an aromatic ring of daunomycin with a terminal G6 quartet by displacing the T7 base, and external groove-binding close to the T1–T2 bases lead to the thermal stabilization of 15 °C, which is likely to inhibit the association of telomerase with telomeres. The findings have implications in the structure-based designing of anthracycline drugs as potent telomerase inhibitors.

## 1. Introduction

Guanine (G) repetitive DNA form a four-stranded non-canonical G-quadruplex DNA structure by Hoogsteen hydrogen bonding. The folding of single-stranded telomeric DNA into a quadruplex structure inhibits telomerase enzyme, affecting cell death by apoptosis pathways. Telomerase expression levels correlate with cancer progression and the metastatic state, and its regulatory potential toward cancer cell growth has been substantiated [1,2]. Besides telomeres, guanine-rich sequences are located in many other biologically significant regions of genomes, such as promoters of oncogenes (e.g., c*-kit*, *c-myc*, *bcl-2*), recombination hot spots, 5′UTR regions, immunoglobulin switch regions; and are correlated with gene expression [3]. Ligands that bind to G-quadruplex and stabilize its structure interfere with telomerase function, oncogene expression, and genomic stability. This has since evolved as an effective strategy and paved the way for the discovery of novel small molecules/ligands that can bind selectively to G-quadruplex DNA. The focus of research in this direction is to understand the mode of ligand–DNA interaction, modify the functional groups, and design potential G-quadruplex binders that have high affinity as well as selectivity over duplex DNA, and in doing so could act as anticancer drugs.

**Figure 1:**
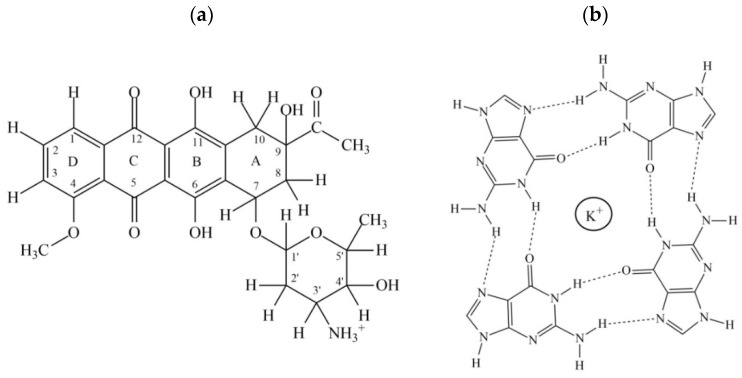
(**a**) Chemical structure of daunomycin and; (**b**) G-tetrad in the presence of K^+^ ions.

A large number of compounds e.g., alkaloids, flavonoids, porphyrins, anthraquinones, anthracyclines, etc. are being evaluated [1,4]. Among anthracyclines, daunomycin (Figure 1a) and doxorubicin, which are used in chemotherapy, are known to interact with duplex DNA by intercalation between base pairs, block DNA/RNA synthesis, and act as topoisomerase II poison [5,6]. They cause considerable DNA damage, cardiotoxicity through free radical generation [6], and the loss of higher-order chromatin structures [7]. Their role in the maintenance of telomeres has recently been evidenced [8,9,10,11,12,13]. Adriamycin treatment in breast cancer cells is caused by an increase in the activity of tumor suppressor protein p53, a decrease in telomerase enzyme activity, and an increase in β-galactosidase [8], thereby inducing senescence. Daunomycin causes an elevation in endogenous ceramide levels, resulting in an 85% inhibition of telomerase in lung cancer cells [9], which is apparently due to a decrease in the level of the *c-myc* transcription factor and the telomerase reverse transcriptase h-TERT protein. The expression of dominant-negative human catalytic subunit of telomerase (DN-hTERT) in the telomerase-positive human acute lymphoblastic leukemia cell line was found to significantly enhance daunomycin-induced apoptosis [10]. Nemorubicin, which is a daunomycin derivative, requires an intact nucleotide excision repair system to exert its activity [11]. Anthracycline treatment also induces telomere dysfunction by suppressing telomerase association with telomeres in MCF7 and HepG2 cancer cell lines by downregulating PinX1 expression [12]. Adriamycin influences the expression of immunomodulatory genes by inducing the secretion of tumor necrosis factor and γ-interferon [13]. This demonstrates that anthracyclines follow multiple strategies of action by targeting different forms of DNA, and interfere with complex mechanisms involved in gene functioning. Competition dialysis showed that daunomycin binds to G-quadruplex DNA [14] beside GC-rich duplex DNA.

G-quadruplex structures formed by human or *Tetrahymena* telomeric DNA repeats, (TTAGGG)_n_ and (TGGGGT)_n_, respectively, can form intermolecular or intramolecular complexes depending upon the sequence length and environmental conditions such as the nature of cations and their concentration [15,16,17,18]. Long telomeric sequences usually exist in several conformations involving different folded forms with different topologies as well as intermolecular aggregates in equilibrium, making ligand–intramolecular G-quadruplex interactions difficult to interpret by structural techniques. Therefore, intermolecular G-quadruplex [d-(TTGGGGT)]_4_ and similar sequences are a viable option for structural studies as a model system [19,20,21,22,23,24,25,26]. Although the simplified model does not contain loops, it contains a similar G-tetrad surface and grooves to biologically relevant G-quadruplexes such as that in antiparallel and hybrid G-quadruplexes; so, despite their limitations, the studies on model systems are found to be informative. Besides, intermolecular types of structures have been found to occur in vivo in recombination, telomere pairing, etc. [27,28]. Therefore, binding sites on G-quadruplex DNA apart from loops are relevant to ligand–G-quadruplex interactions. Electron spray ionization mass spectrometry showed [29] that daunomycin binds to [d(TTGGGGGT)]_4_ and the collision-induced dissociation of daunomycin-[d-(TGGGGT)]_4_ complex [30] occurs via the loss of ligand leaving the intact G-quadruplex. The first X-ray crystallographic structure of daunomycin-[d-(TGGGGT)]_4_ complex [23] showed two layers of daunomycin containing three molecules each, which were sandwiched between two G-quadruplexes with daunosamine sugar moiety inserted in grooves, while another daunomycin-d-(GGGG)]_4_ complex indicated the absence of any such interaction [24]. Molecular Dynamics (MD) simulations of the daunomycin-d-(TGGGGT)_4_ [25] complex, on the contrary, showed that daunomycin binds in a monomeric state through stacking interactions with the last G-quartet as well as at grooves, both with practically the same binding affinity. Nuclear magnetic resonance (NMR) studies on the interaction of daunomycin analogues, nemorubicin and doxorubicin, with G-quadruplex sequences containing three guanine repeats, e.g., [d-(TTAGGGT)]_4_, showed [26] that binding takes place at A3pG4 and terminal G-tetrad. There are no investigations of absorption, fluorescence, or circular dichroism spectroscopy techniques that could independently provide any evidence of mode of interaction. Further, corresponding thermal melting profiles have also not been reported in the literature, which could substantiate that binding directly causes an enhancement in the stability of DNA, which is a major therapeutic index for G-quadruplex-based ligands as drugs, although these techniques have been used for some hybrid/basket conformations in 21/22-mer DNA quadruplexes [31,32,33].

Daunomycin lacks selectivity of binding to a DNA quadruplex over a DNA duplex. While attempting to design G-quadruplex ligands as successful drugs, an understanding of a molecular basis of interactions in non-selective drugs that bind to both duplexes as well as quadruplexes, is essential. We have undertaken a study of the interaction of daunomycin with [d(TTGGGGT)]_4_ (G-quartet as in Figure 1b) by various biophysical techniques. We show real-time binding by Surface Plasmon Resonance (SPR) experiments for the first time. The stoichiometry of complexes is determined independently by Job Plot using fluorescence. Circular Dichroism (CD) spectra reveal conformational aspects of binding. Diffusion Ordered SpectroscopY (DOSY) experiments confirm the formation of a stable complex. Detailed analysis of proton NMR spectra, including direct short interproton contacts in Nuclear Overhauser Enhancement SpectroscopY (NOESY) spectra, give valuable information on specific interactions at the molecular level. Phophorus-31 NMR provide information on DNA backbone geometry. The thermal melting profiles of imino protons have been examined and correlated to Differential Scanning Calorimetry (DSC) thermograms. The findings confirm significant thermal stabilization due to the direct binding of daunomycin to G-quadruplex DNA.

## 2. Results and Discussion

### 2.1. Surface Plasmon Resonance, Job Plot, Circular Dichroism, and Diffusion Ordered Spectroscopy

**Figure 2:**
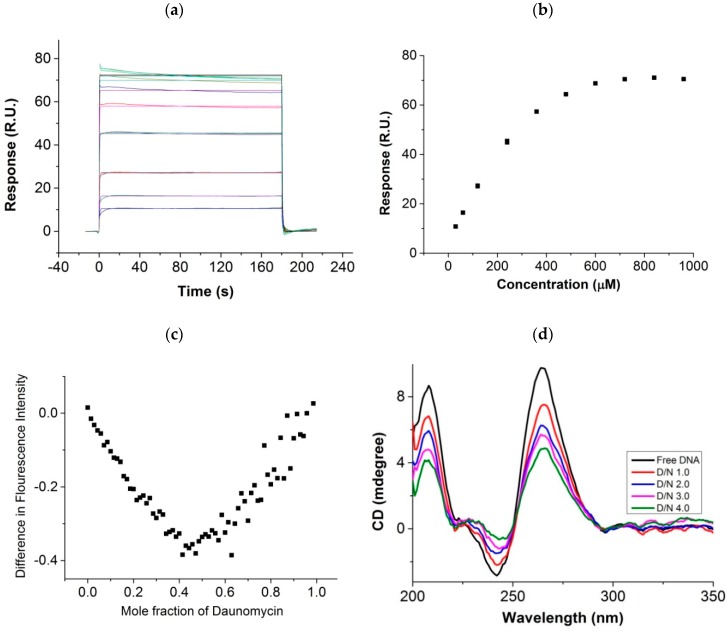

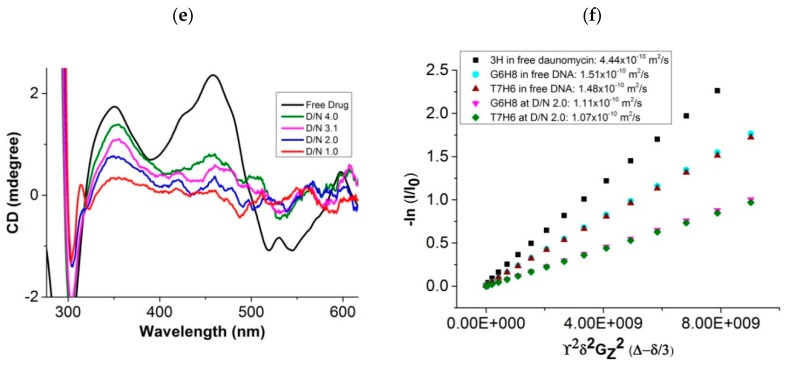
(**a**) Results of surface plasmon resonance experiments for the binding of daunomycin to [d-(TTGGGGT)]_4_. Sensograms obtained for free daunomycin concentration from 30 µM (bottom) to 960 µM (top), using HEPES buffer with 100 mM of KCl at 25 °C; (**b**) Binding plot of steady-state response (R.U.) versus concentration of daunomycin (µM); (**c**) Job Plot for binding of daunomycin to [d-(TTGGGGT)]_4_ showing a difference in the fluorescent intensity of bound and unbound daunomycin as a function of the mole fraction of daunomycin. The total concentration of daunomycin and [d-(TTGGGGT)]_4_ was kept constant as 7 µM in 10 mM of phosphate buffer (KBPES) containing 100 mM of KCl at 25 °C; (**d**) Circular dichroism spectra of 15 µM of free [d-(TTGGGGT)]_4_ and its complex at Daunomycin (D) to Nucleic acid (N) ratio, D/N = 1.0, 2.0, 3.0, and 4.0; (**e**) Circular dichroism spectra of 400 µM of free daunomycin and its complex with [d-(TTGGGGT)]_4_ at D/N = 1.0, 2.0, 3.0, and 4.0 in 10 mM of phosphate buffer containing 100 mM of KCl at 25 °C; and (**f**) Diffusion coefficient measurement obtained from Diffusion Ordered SpectroscopY (DOSY) spectra of free daunomycin, free [d-(TTGGGGT)]_4_, and daunomycin-[d-(TTGGGGT)]_4_ complex at D/N = 2.0 at 25 °C.

The surface plasmon resonance sensograms (Figure 2a) give a direct proof of binding of daunomycin with [d-(TTGGGGT)]_4_. The steady-state response increases with concentration in the range between 30–960 μM (Figure 2b), indicating that a specific interaction of daunomycin with [d-(TTGGGGT)]_4_ indeed does take place. The binding isotherms yield the affinity constant K_b_ ~4.1 × 10^3^ M^−1^ at 25 °C, referring to the dominant mode of interaction. The direct assessment of the stoichiometry of complex made by continuous variation (Job Plot) analysis using fluorescence shows a slope change between approximately linear regions at a mole fraction of daunomycin of ~0.50 and 0.66, yielding stoichiometry ratios of ~1:1 and 2:1 for the daunomycin:[d-(TTGGGGT)]_4_ complex (Figure 2c). We observed a scatter of data at mole fraction 0.5–0.7, which may be due to the existence of multiple stoichiometric complexes, although the highest stoichiometry attained in the complexes appears to be 2:1. The circular dichroism spectra of 15 μM uncomplexed [d-(TTGGGGT)]_4_ showed (Figure 2d) two well-defined signature peaks of right-handed parallel-stranded quadruplex DNA around 264 nm and 242 nm [18], which are characteristic of the stacking pattern and helicity, respectively. The observed decrease in the intensity of CD bands on the addition of varying concentrations of daunomycin (D) to a fixed concentration of nucleic acid (N) at the mole equivalent ratios, D/N = 1.0–4.0, confirm an interaction between the two molecules without any significant alteration in the shape and position of the CD bands. The CD measurements reflect on the mode of interaction between two interacting molecules. Groove-binding has been shown to induce changes in the magnitude of 265 nm and 243 nm bands, while end-stacking does not show any significant change [34]. Accordingly, both end-stacking and external groove-binding may be occurring in present investigations. The stacking pattern of G quartets does not change appreciably, but the overall conformation of tetra molecular parallel-stranded [d-(TTGGGGT)]_4_ is less helical upon binding; that is, helicity appears to be relaxed without being entirely lost. Alternately, we titrated a fixed concentration of 0.4 mM of daunomycin with varying concentrations of [d-(TTGGGGT)]_4_ at D/N = 1.0–4.0 (Figure 2e). Free daunomycin shows characteristic bisignate CD bands with a positive band at 460 nm, a negative band at 500 nm, and crossover at 540 nm [35], which refers to the dimer formation at such high concentrations [36,37,38]. The addition of DNA completely disrupts the daunomycin dimer, and rules out the possibility of daunomycin molecules binding as a stacked dimer in any parallel or antiparallel orientation to DNA.

The ^1^H DOSY experiments showed (Figure 2f) that a diffusion constant of 1H/2H/3H protons (~4.0 × 10^−10^ m^2^/s) in free daunomycin and T7H6/G6H8 protons in free DNA (~1.5 × 10^−10^ m^2^/s) are greater than that for bound DNA (~1.0 × 10^−10^ m^2^/s) at D/N = 2.0 at 25 °C. This shows that ligand-bound DNA undergoes diffusion at a slower rate as a stable complex upon binding [21,39]. Due to the presence of two stoichiometries as evidenced by Job Plot and perhaps two binding sites, daunomycin proton signals were broadened and could not be used for calculating diffusion constants. The DOSY spectra thus confirm the formation of a stable bound complex.

### 2.2. Proton Nuclear Magnetic Resonance

The complete unambiguous assignment of all of the exchangeable and non-exchangeable protons in free [d-(TTGGGGT)]_4_ (Figure S1a,b) was made (Table 1 and Table S1a,b) by rigorous analysis of two-dimensional (2D) ^1^H-^1^H NOESY, ^1^H-^1^H COSY, and ^1^H-^13^C HSQC spectra [39,40,41]. The presence of imino signals of four guanines protons at 10.5–11.5 ppm show the presence of Hoogsteen base pairing among G quartets, while G-quadruplex formation is further ascertained by NOE (Nuclear Overhauser Enhancement) correlations between adjacent GNH protons and sequential NOEs between GNH (Figure S1c) and preceding GH8/TH6/TCH_3_ protons (Figure S1d). A single set of GNH, GH8, TH6, and TCH_3_ in every G-tetrad showed fourfold symmetry of quadruplex [d-(TTGGGGT)]_4_. Intranucleotide and sequential internucleotide NOE correlations, between base H8/H6 and deoxyribose H1′/H2′/H2′′ protons (Figure S1e,f), confirm the existence of a tetramolecular parallel quadruplex having right-handed B-DNA conformation with predominant C2′ endo sugar pucker and anti glycosidic bond rotation [27,39,40,41]. The imino protons of thymine were in fast exchange with solvent, and could not be ascertained for hydrogen bond formation and the existence of a tetrad [22].

**Figure 3:**
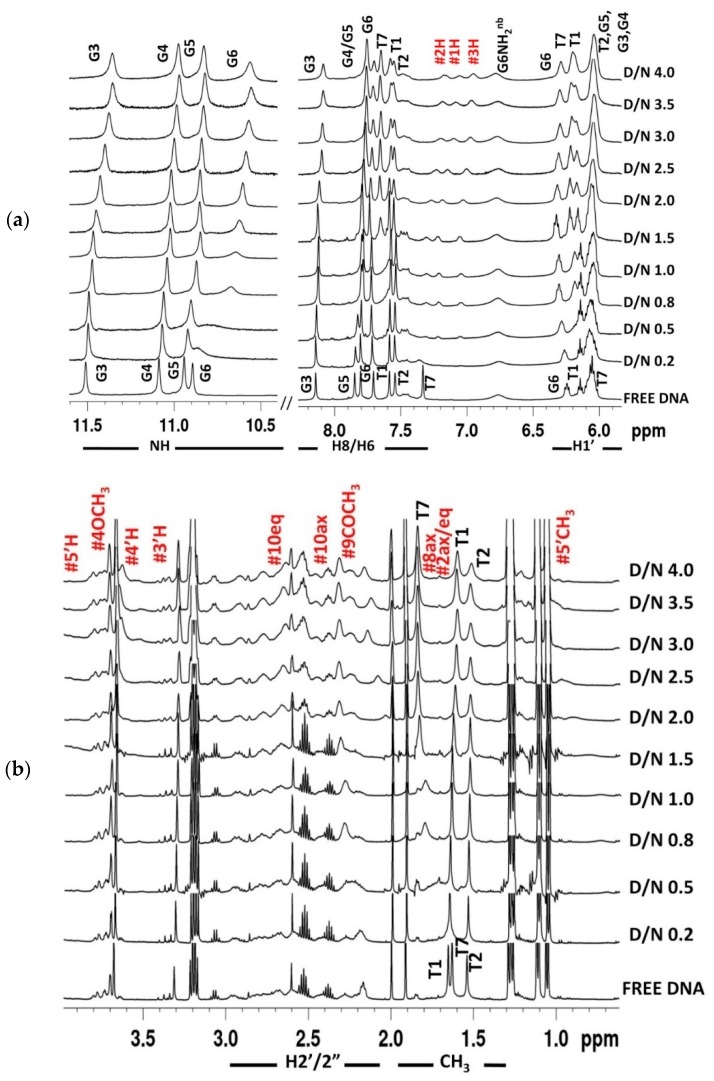
1D ^1^H NMR spectra of 1.16 mM of [d-(TTGGGGT)]_4_ and its complex upon the progressive addition of daunomycin to DNA at different D/N ratios at 25 °C. (**a**) Imino, base, and sugar H1′ protons along with ring D aromatic protons 1H, 2H, and 3H of daunomycin; (**b**) Deoxyribose sugar H2′/2′′ and methyl protons of thymine along with daunomycin protons 5′H, 4OCH_3,_ 4′H, 3′H, 10eq, 10ax, 9COCH_3_, 8ax, 2ax, 2eq, and 5′CH_3_ at 25 °C. Symbol # denotes daunomycin protons.

The quadruplex [d-(TTGGGGT)]_4_ was titrated with daunomycin at D/N = 0.0–4.0 and monitored by ^1^H and ^31^P NMR at several D/N ratios at three different temperatures: 25 °C, 30 °C, and 40 °C. Two-dimensional ^1^H-^1^H NOESY, ^1^H-^13^C HSQC, and ^1^H-^31^P HMBC spectra were recorded at D/N = 1.0, 2.0, 3.0, and 4.0 at 25 °C. ^1^H-^1^H NOESY and ^1^H-^13^C HSQC spectra were also recorded at 35 °C at D/N = 1.0, 2.0, and 3.0. The progressive addition of daunomycin to 1.16 mM of [d-(TTGGGGT)]_4_ resulted (Figure 3a,b) in the shifting and broadening of DNA proton signals along with the appearance of new signals, which were subsequently assigned to daunomycin protons. A single set of sharp, well-defined and intense resonances of GNH, GH8, TH6, and TCH_3_ protons shows that there is no loss of fourfold symmetry of [d-(TTGGGGT)]_4_ upon complexation. The unambiguous assignment of all non-exchangeable and exchangeable protons of [d-(TTGGGGT)]_4_, as well as daunomycin protons in complex at D/N = 1.0–4.0, was accomplished (Table 1, Table 2, Table S1a–c and Table S2a–d) by an analysis of proton NMR spectra of free [d-(TTGGGGT)]_4_ (Figure S1a,b), free daunomycin (Figure S2), the daunomycin-[d-(TTGGGGT)]_4_ complex (Figure S3a,b), their corresponding ^1^H-^1^H NOESY (Figure S1c–f or Figure S3c–f), and the ^1^H-^13^C HSQC (Figure S4a–d) spectra. The resonance signals of deoxyribose protons of DNA could be clearly distinguished from the daunosamine sugar and ring A/D protons signals in ^1^H-^13^C HSQC spectra (Figure S4a–d), in spite of spectral overlap. The 1H and 3H of daunomycin (7.0–7.3 ppm) show clear distance correlation with 4OCH_3_ resonance (3.67 ppm). The carbons attached to them resonate at ~119 ppm (Figure S4a), which is far apart from the ^13^C signals of the bases (133–139 ppm) and deoxyribose H1′ (82–92 ppm) (Figure S4c). The ^13^C signals (63–67 ppm) of deoxyribose H5′/5′′ (3.7–4.4 ppm) separate out from those of 4OCH_3_, 7H, 5′H, 4′H, and 3′H protons (Figure S4b). Similarly, 2axH, 2eqH, 10axH, 10eqH, 8axH, 8eqH, and 9COCH_3_ resonances (1.7–2.8 ppm) that overlap with deoxyribose H2′/H2′′ (2.2–3.0 ppm) have ^13^C signals close to that for free drugs in the range 22–35 ppm (Figure S4d), which are well separated from the corresponding ^13^C signals of H2′/H2′′ (36–44 ppm). The existence of a single set of resonances for both ligand and DNA show that a well-defined stable complex is formed, which refers to the major conformer present in the ligand–DNA complex.

**Figure 4:**
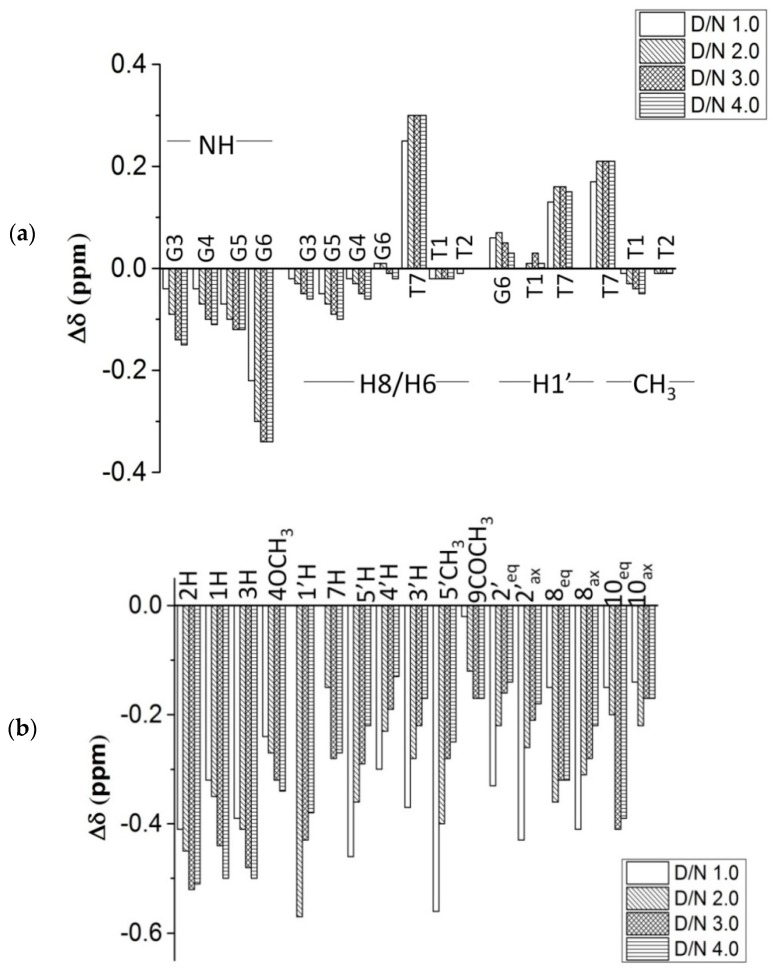
Bar diagram showing change in ^1^H chemical shift (Δδ) of (**a**) [d-(TTGGGGT)]_4_ protons and (**b**) daunomycin protons on complexation at D/N = 1.0, 2.0, 3.0 and 4.0 in 10 mM phosphate buffer with 100 mM of KCl (90% H_2_O + 10% D_2_O) at 25 °C.

The initial addition of 0.2 and 0.5 mole equivalents of daunomycin to [d-(TTGGGGT)]_4_ resulted in the severe broadening of G6NH, T7H6, and T7CH_3_ (Figure 3a,b), which was suggestive of binding close to these bases. The broadening is at a maximum at D/N = 1.0. Upon the addition of two mole equivalents of the drug, all three resonances become sharp and remain sharp up to D/N = 4.0, pointing toward a stoichiometry of 2:1 in daunomycin-[d-(TTGGGGT)]_4_ complex [20,21,22,26,39,40,41]. This is in accord with the findings by Job Plot, which uses lower concentrations of DNA but identical conditions of salt concentration and temperature. The patterns of perturbations in NMR spectra reflect the position, stability, and kinetics of binding events. The G6NH, T7H6, T7CH_3_, and T7H1′ show continuous changes in chemical shifts up to D/N = 2.0–2.5, beyond which they stop drifting (Table S2a–c). On the other hand, T1CH_3_, G3NH, G3H8, and G4H8 show a noticeable upfield shift (0.05–0.15 ppm) (Table S2a–c), as well as a broadening of signals (Figure 3a,b) at D/N > 2.0–2.5. Therefore, another binding site is located close to the T1-T2-G3 bases on the 5′-end. The binding at two sites may be sequential, or else simultaneous binding takes place at two sites, but binding affinity at the T1-T2-G3 site is apparently lower, so that the site gets occupied at relatively higher D/N ratios. G6NH shifts significantly upfield by 0.34 ppm, while T7H6, T7CH_3_, and T7H1′ shift downfield by 0.30 ppm, 0.21 ppm, and 0.15 ppm, respectively. Sequence-specific changes in the proton NMR signals of DNA rule out the existence of a non-specific adherence of daunomycin externally to DNA. Changes in chemical shift reflect the chemical environment of interacting molecules, and a large upfield shift is generally indicative of stacking interactions [26,39,40,41,42,43]. Apparently, the G6 base is better stacked on complexation, while T7 appears to be destacked. This can occur if the ligand stacks with a G6 quartet and a T7 unit, which is rather flexible, is pushed away by the ligand. The change in the chemical shift in T1, T2, and G3 protons is relatively much lesser (Figure 4a), being a maximum of 0.15 ppm in G3NH. It is possible that ligand binds near this site externally without significantly affecting DNA conformation. Also, the G4 and G5 residues show minor changes throughout the course of titrations, which may be due to the minor conformational adjustments in DNA as more and more daunomycin binds. We obtained an estimate of the binding constant from the variation in chemical shift of several protons with a D/N ratio (Table S2a–c) that gives K_b_ ~1.8–2.3 × 10^4^ M^−1^ for T7H6 and T7CH_3_ protons, 2.3 × 10^3^ M^−1^ for G6NH protons, and 1.4 × 10^2^ M^−1^ for T1CH_3_ protons, (Figure S5a–d), so that binding at G6-T7 residues has greater affinity than the corresponding T1-T2-G3 site. Daunomycin protons show expected intramolecular NOE connectivities (Table S3) due to the proximity of pairs of protons, as observed in case of free daunomycin [37]. All of the daunomycin protons shift upfield upon binding to DNA. The upfield shift in 2H, 1H, 3H, 4OCH_3_, 1′H, 8eqH, and 10eqH are significant, being 0.51 ppm, 0.50 ppm, 0.50 ppm, 0.34 ppm, 0.38 ppm, 0.32 ppm, and 0.39 ppm, respectively, while other protons shift by 0.13–0.27 ppm at D/N = 4.0 (Figure 4b, Table 2). The change is continuous with a D/N ratio in the range D/N = 0.5–4.0. Since the binding affinity at one site is relatively stronger, both sites may not be occupied at D/N = 2.0. The aromatic ring protons of ring D and those lying equatorially or close to plane ABCD of the aromatic rings are upfield shifted to a large extent, which is perhaps due to the ring current effects of nucleic acid bases. Since all of the sequential NOE connectivities are intact and there is no possibility of classical intercalation, the ABCD ring of daunomycin could partially stack with the G6 base by destacking the T7 base. Among the daunosamine sugar protons, significantly large upfield shifts in 5′H and 5′CH_3_ protons (0.27–0.31 ppm) showed that daunosamine sugar may interact with the DNA backbone in the bound complex. Although DNA peaks sharpened at D/N = 2.0, the daunomycin peaks remained broader than the DNA base and imino signals. The existence of only one set of daunomycin signals requires the ligand to be in fast exchange among all of the possible bound conformations; that is, due to either of the two sites or both DNA sites being occupied by the ligand. The hydroxyl protons at the 6, 11, 9 and 4′ positions were not observable in any spectra of complex, even at low temperatures, that is, 5 °C.

### 2.3. Phosphorus-31 Nuclear Magnetic Resonance

**Figure 5:**
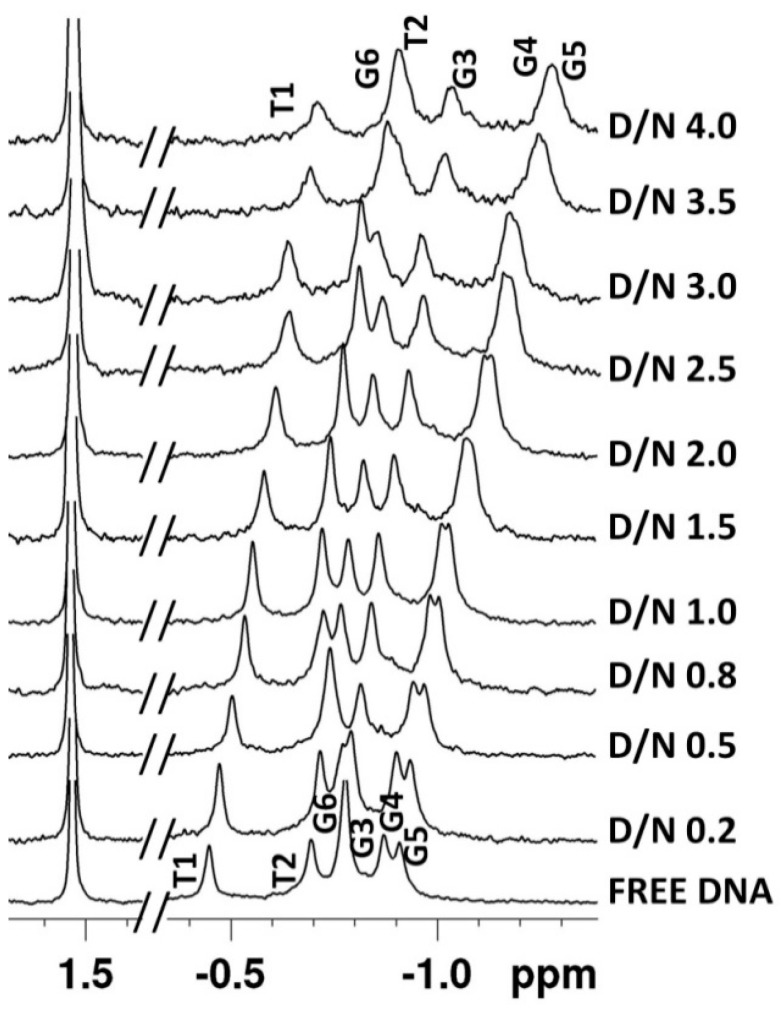
Proton decoupled ^31^P NMR spectra of 1.16 mM [d-(TTGGGGT)]_4_ and its complex upon the progressive addition of daunomycin at different D/N ratios in 10 mM of phosphate buffer with 100 mM of KCl (90% H_2_O + 10% D_2_O) at 25 °C.

The phosphorus-31 NMR signals were assigned using standard strategies by ^1^H-^31^P HMBC spectra using ^3^*J* and ^4^*J* coupling with (H3′)_n_/(H5′/H5′′)_n+1_ and (H4′)_n_/(H4′)_n+1_, respectively in free (Figure S6a) and complexed DNA (Figure S6b) at 25 °C [39,44,45]. Upon the progressive addition of daunomycin, the ^31^P signal of G6pT7 shifted slightly downfield up to D/N = 1.0, and then upfield up to D/N = 4.0, while all of the other ^31^P signals shifted gradually upfield (Figure 5, Table S4). The drift in resonances is sequence-dependent, it being 0.27 ppm, 0.23 ppm, 0.25 ppm, 0.38 ppm, 0.39 ppm, and 0.13 ppm upfield for T1pT2, T2pG3, G3pG4, G4pG, G5pG6, and G6pT7, respectively at D/N = 4.0, which is indicative of the specificity of interaction between daunomycin and the G-quadruplex. It is known that ^31^P chemical shifts vary in response to the local sequence-specific distortions induced in DNA duplex geometry [46]. Theoretical and experimental studies in duplex DNA have shown [45,46] that switching from energetically more favorable B_I_ conformation (ζ = g^−^, α = g^−^) to a more flexible B_II_ conformation (ζ = t, α = g^−^) with 1 Kcal/mole greater energy induces a downfield shift ~1.5–2.0 ppm, which is required to open the base pairs from 3.4 Å to 6.8 Å to permit intercalation between two base pairs. Besides, the widening and narrowing of the ester O-P-O bond angle due to distortions caused in the DNA backbone lead to small upfield and downfield shifts, respectively. Purely electrostatic interactions between the drug and the DNA in drug–DNA complexes produce small upfield shifts. The dispersion in ^31^P chemical shifts may also result from different populations of B_I_ and B_II_ conformational states [45,46]. The observed upfield shifts may therefore be attributed to the proximity of the 3′NH_3_ moiety to phosphates of DNA or the widening of the ester bond. The absence of a large downfield shift completely rules out the opening of base pairs to allow classical intercalation at any step along the DNA. These results clearly prove that daunomycin binds to d-(TTGGGGT)]_4_ externally, and certainly not by the classical intercalation mode of binding.

### 2.4. NOE Correlations and Conformation of Complex

**Figure 6:**
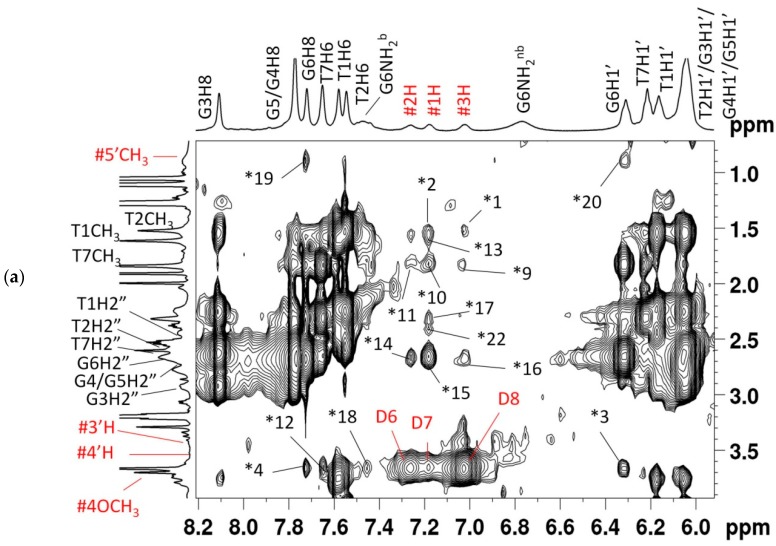

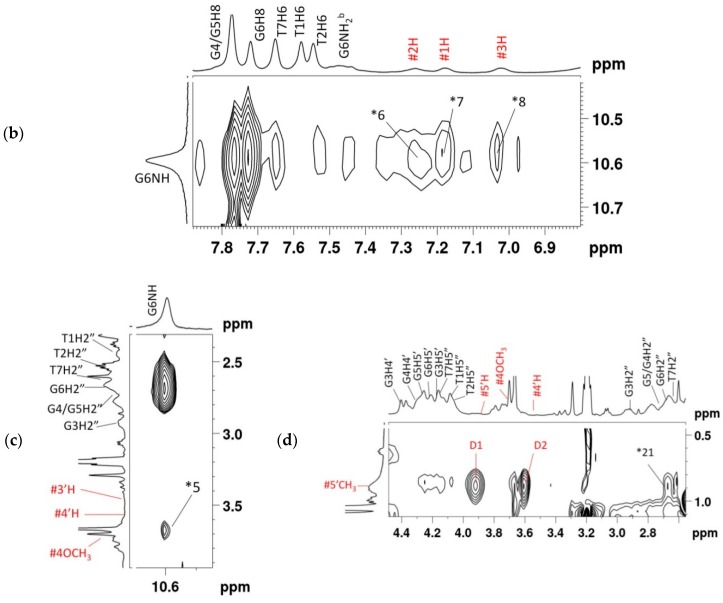
2D ^1^H-^1^H Nuclear Overhauser Enhancement SpectroscopY (NOESY) spectra of daunomycin-[d-(TTGGGGT)]_4_ complex at D/N = 2.0, mixing time τ_m_ = 250 ms, at 25 °C. Expansion of specific regions of NOESY spectra showing NOE (Nuclear Overhauser Enhancement) correlations between (**a**) daunomycin protons 1H, 2H, 3H, 5′CH_3_, 3′H, 4′H, and 4OCH_3_, and H8/H6/H1′/H2′/H2′′/CH_3_ protons along with intramolecular NOE cross-peaks of daunomycin (D); (**b**) Daunomycin protons 1H, 2H, 3H, and imino proton, (**c**) daunomycin proton 4OCH_3_ and imino proton; and (**d**) Daunomycin proton 5′CH_3_ and sugar H2′′ proton along with intramolecular NOE cross-peaks of daunomycin (D). Symbol # denotes daunomycin protons, D denotes intramolecular cross-peaks of daunomycin (D numbering as in Table S3), and * denotes intermolecular cross-peaks between daunomycin and DNA [d-(TTGGGGT)]_4_ protons (* numbering as in Table 3).

The sequential NOE cross-peaks among adjacent guanine imino protons and imino to base protons (Figure S3c,d) confirm the continuity between guanine bases forming G-quartets, and that the G-quadruplex DNA structure is intact after binding to daunomycin. The entire pattern of classical sequential connectivities of base protons to sugar H1′/H2′/H2′′ protons of neighboring bases at the 5′ end as well as among intranucleotide protons shows that the conformation of daunomycin-[d-(TTGGGGT)]_4_ complexes closely resemble that of free [d-(TTGGGGT)]_4_ having right-handed helix and anti glycosidic bond rotation in all guanines. The presence of all sequential NOEs of H8/H6 with H1′/H2′/H2′′ (Figure S3e,f) indicates that there is no opening of base pairs from 3.4 Å to 6.8 Å at any 5′–3′ base pair step to permit a classical kind of intercalation of the aromatic chromophore of daunomycin between base pairs, as observed in the case of both X-ray crystallographic [47] and NMR solution structures [42,45] of complexes of daunomycin with duplex DNA sequences [d-CGATCG]_2_, [d-TGATCA]_2_, [d-TGTACA]_2_, etc. This gives independent evidence of the absence of any possibility of classical drug intercalation as a possible mode of interaction in a DNA quadruplex. Eleven intramolecular NOE cross-peaks that were expected due to short distance contacts within daunomycin were observed (Figure 6a or Figure S7, Table S3). In addition, 22 intermolecular NOE cross-peaks between daunomycin and T1, T2, G6, and T7 protons were observed (Figure 6a–d, Table 3), indicating the presence of two distinct binding sites in DNA, that is, T1-T2 and G6-T7, corroborating the stoichiometry of 1:1 and 2:1 determined independently by Job Plot. The intermolecular NOEs between ring D aromatic 1H, 2H, 3H, and 4OCH_3_ protons with G6NH/G6H8/T7CH_3_/G6H1′ show the stacking of daunomycin with base quartets. The existence of these short distance contacts is in accord with the observed large upfield shifts of up to 0.45 ppm in these protons (Figure 4b, Table 2). Three NOE cross-peaks of 5′CH_3_ of daunosamine sugar with G6H8/G6H1′/G6H2′′ indicate its proximity to DNA, which also showed a large upfield shift ~0.40 ppm as compared to other daunosamine protons. Apart from a large mixing time (400 ms), these NOEs were also seen at a short mixing time (200 ms), so the chances of a combination of transferred NOEs or exchange effect of two orientations of a single molecule can be excluded. There were no detectable NOEs from daunomycin to any part of the interior G4 plane, and this plane therefore appears to have no contact with the ligand. Also, the chemical shift of G3/G4/G5 protons were not significantly influenced by ligand binding, although the G3H8 showed line broadening. We observed some extra resonance peaks of low intensity, particularly in 1.2–1.8 ppm range, which was apparent in NOESY spectra at D/N > 2.0. Some of these signals give weakly intense intermolecular NOEs with ring D protons of daunomycin. Such NOEs, which do not match with the sharp major assigned TCH_3_ peaks, were not considered for analysis. These presumably arise due to a presence of a minor conformation of complex.

**Figure 7:**
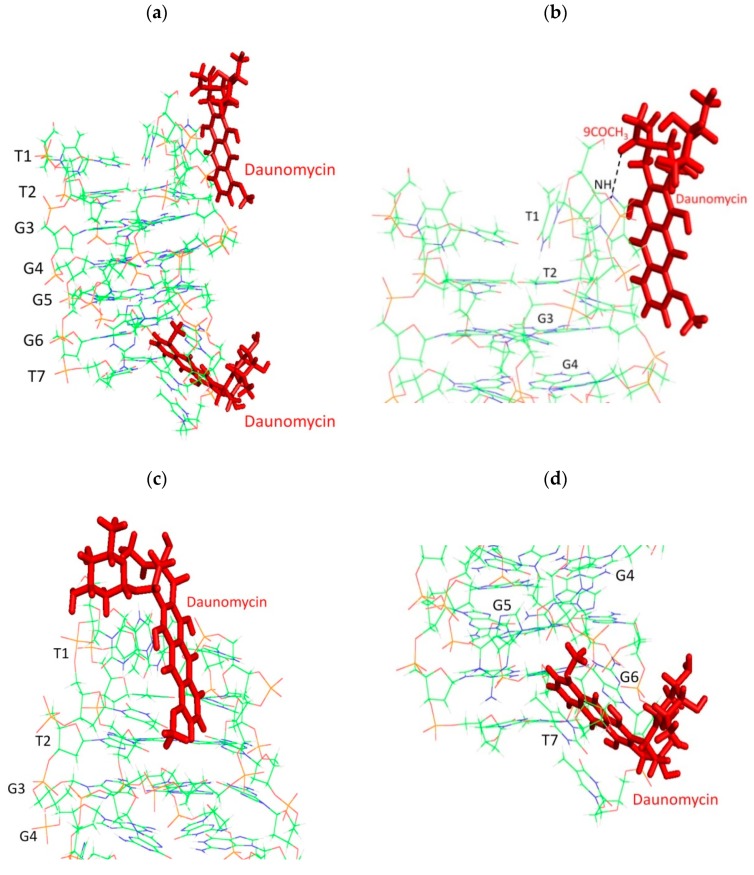

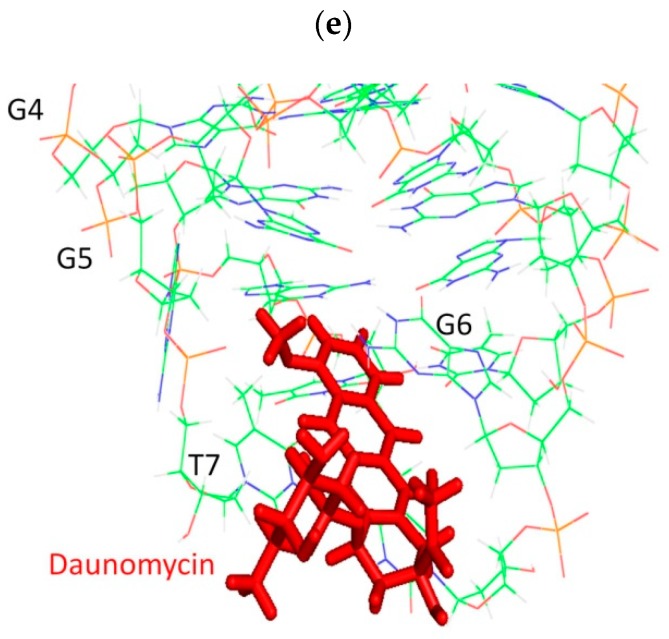
Energy minimized model of daunomycin bound to [d-(TTGGGGT)]_4_ obtained by restrained molecular dynamics simulations; (**a**) Binding of daunomycin at two independent sites T1pT2 and G6pT7; (**b**,**c**) Close-up view of daunomycin binding to [d-(TTGGGGT)]_4_ at the T1pT2 site; and (**d**,**e**) At G6pT7 site (hydrogen bond is represented by black dashed line).

Distance restraints from 13 non-overlapping intermolecular NOEs (Table 3) that were found in NOESY spectra recorded at all mixing times (200–400 ms) and at each D/N ratio were used to build the model by positioning daunomycin at two independent sites close to the T1pT2 and G6pT7 steps of [d-(TTGGGGT)]_4_. The restrained Molecular Dynamics (rMD) simulations approach that was used to arrive at a conformation that fits into observed NOE data included 138 intra-DNA and 11 intra daunomycin (Table S3) distance restraints. Stacking interaction between daunomycin chromophore and G6pT7 bases was evident in the rMD structure (Figure 7a), while external binding was observed at the T1pT2 site. Rings A, B, C, and D of the daunomycin molecule bind at the groove of the T1pT2 base (Figure 7b,c). The ring D of daunomycin stacks with the purine ring of the terminal G6 base by a slight opening of the T7 base, thus bringing 4OCH_3_ close to the G-quartet (Figure 7d,e). A single hydrogen bond was formed between 9COCH_3_ and the NH of the T2 base at the T1pT2 site (Figure 7b). The energy terms, restraints violations, and RMSD (Root Mean Square Deviation) of the obtained structure are reported in (Table 4). Out of the total restraint violations of 3.3% (5 out of 151), 2.6% belonged to the T7 base, so that the impact of the restraint violations was minimal. The electrostatic and van der Waals energy contribution to the total energy suggest a role of stacking and the electrostatic attraction of NH_3_^+^ of the daunosamine sugar moiety to the DNA backbone.

The observed intermolecular short distance NOE contacts between daunomycin and DNA as well as the rMD structure provided direct confirmation of end-stacking and external groove-binding at G6-T7 and T1-T2 sites, respectively. This is at variance with the X-ray crystallographic structure, which showed layers of daunomycin stacked between two quadruplexes [23], but in accord with the MD simulations in explicit solvent for daunomycin-[d-(TGGGGT)]_4_ [25]. The end-stacking mode was also inferred from the collision-induced fragmentation patterns in the daunomycin-[d-(TGGGGT)]_4_ complex [30] and NMR studies on the doxorubicin-[d-(TTAGGGT)]_4_ complex [26]. It has been suggested [26] that crystal-packing forces may be responsible for the observed difference in the structure of the complex. The present NMR experiments and molecular dynamics simulations [25] have been performed in explicit water solvent, which may offer structural flexibility to the DNA.

Further, our NMR results show that daunomycin binds as a monomer at grooves or stacks with terminal G-quartets, unlike the X-ray crystallographic structure of the daunomycin-[d-(TGGGGT)]_4_ complex [23], in which two layers of daunomycin containing three drug molecules each are sandwiched between two G-quadruplexes stacked end-to-end. Two daunomycin molecules oriented in antiparallel orientation with respect to each other are expected to give intermolecular NOE cross-peaks due to the stacking of ring A protons with ring D; that is, 10axH/10eqH with 1H/2H/3H/4OCH_3_, 8axH/8eqH with 1H/2H/3H/4OCH_3_, 9COCH_3_ with 2H/3H, 7H with 1H/2H/3H, and 5′CH_3_ with 1′H/2′H/3′H [37,38]. On the other hand, stacking between two daunomycin molecules oriented parallel to each other is expected to show NOE cross-peaks of ring D protons with daunosamine sugar protons; that is, 10axH/10eqH with 4′H/3′H/5′H/5′CH_3_, and 10axH/10eqH with 7H, 9COCH_3_ with 3′H/5′H [38]. Some of these short contacts are evident in X-ray crystal structures [23]. The NOEs of ring A protons of one daunomycin molecule with ring A protons of a second daunomycin stacked over it in parallel orientation are relatively weaker in intensity than the corresponding intramolecular NOE connectivities, so that the intensity of intramolecular NOE cross-peaks will dominate in NOESY spectra, and cannot be used to judge parallel orientation. We did not observe any drug–drug intermolecular NOE cross-peak at any D/N ratio in the NOESY spectra of complexes in the present investigations. Hence, neither a parallel/anti parallel dimer of daunomycin binds, nor do two layers of daunomycin molecules sandwiched between two G-quadruplexes, exist in the solution structure of a complex, as observed in the crystal structure of the daunomycin-[d-(TGGGGT)]_4_ complex [23]. Besides, the stoichiometry of complex in a crystal structure [23] would be 3:1, while our Job Plot shows (as seen in Figure 2c) that maximum stoichiometry is reached is 2:1. In addition, the observed CD spectra did not exhibit any bisignate peaks (Figure 2e), which is an independent proof of the non-existence of stacked layers of daunomycin or the presence of daunomycin dimer in parallel or antiparallel orientation in our solution studies. This is in accordance with the findings by rMD simulations of daunomycin-[d-(TGGGGT)]_4_ [25] complexes, in which only a monomer is found to bind to DNA.

### 2.5. Thermal Denaturation

**Figure 8:**
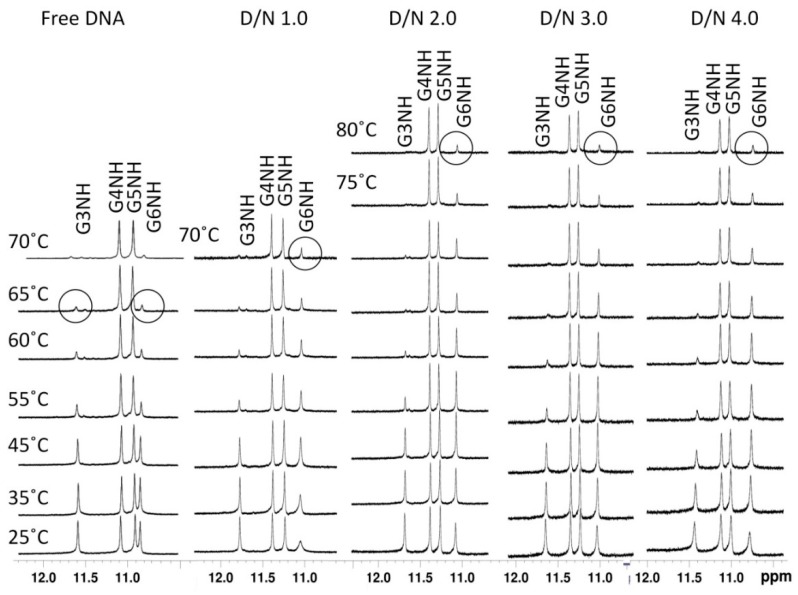
One-dimensional (1D) ^1^H NMR spectra of imino protons showing the thermal melting profile of the free [d-(TTGGGGT)]_4_ and daunomycin [d-(TTGGGGT)]_4_ complex at different D/N ratios and different temperatures.

The melting profiles of GNH protons in free and bound DNA at D/N = 1.0–4.0 were obtained at 25–80 °C in steps of 5 °C upon its transition from an ordered to a disordered state. (Figure 8). In free [d-(TTGGGGT)]_4_, G3NH and G6NH signals appear as low-intensity resonances at a temperature ~65 °C due to the breaking of Hoogsteen hydrogen bonds between guanines on a quartet plane, and the exchange of the NH proton with solvent. However, G4NH and G5NH exist as sharp well-defined resonances at 65–80 °C, so both G3NH and G6NH disappear first (T_m1_ > 65 °C) followed by G4NH and G5NH (T_m2_). The melting of free [d-(TTGGGGT)]_4_ apparently follows a three-state process (alternately two “two-state” melting processes) in which an intermediate conformation of G-quadruplex DNA exists that has G3NH, G6NH in unfolded form, and G4NH and G5NH as still folded, so that T_m1_ ~65 °C (±5 °C). Meanwhile, T_m2_ may be comparatively quite high, but this could not be ascertained, as data was not recorded beyond 80 °C [39,41]. T_m_ decreases in the order: G4NH, G5NH > G6NH, G3NH. On complex formation, imino protons are stabilized to higher temperatures (Figure 8). The G3NH signal is of a relatively lower intensity at D/N = 1.0–4.0 as compared to the corresponding G6NH signal. G3NH disappears at D/N = 1.0, 2.0, 3.0, and 4.0 at about >70 °C, >75 °C, >80 °C, and >80 °C, respectively, so that T_m1_ increases with molar ratio. G6NH disappears at temperature >80 °C at D/N = 1.0–4.0. The melting transitions therefore follow a four-state process in which first G3NH disappears (T_m1_ ~80 °C), and then G6NH disappears (T_m2_ > 80 °C), followed by the G4NH and G5NH protons, which appear as sharp resonances at 80 °C, indicating that T_m3_ may be quite high. Thus, T_m_ decreases in the order: G4NH, G5NH > G6NH > G3NH. The terminal G-quartets, involving G3NH and G6NH, are shielded upon binding to daunomycin, which thermally and efficiently stabilizes the tetramolecular G-quadruplex DNA. G6NH, which was disappearing along with G3NH in free form, is stabilized upon complex formation to a much greater extent than G3NH. This enhanced stabilization is consistent with an observed large upfield shift and the significant broadening of G6NH, T7H6, and T7CH_3_ as compared to all other protons, establishing G6pT7 as the major binding site involving π–π stacking interactions with a daunomycin aromatic ring, leading to an estimate of thermal stabilization of ~15 °C.

**Figure 9:**
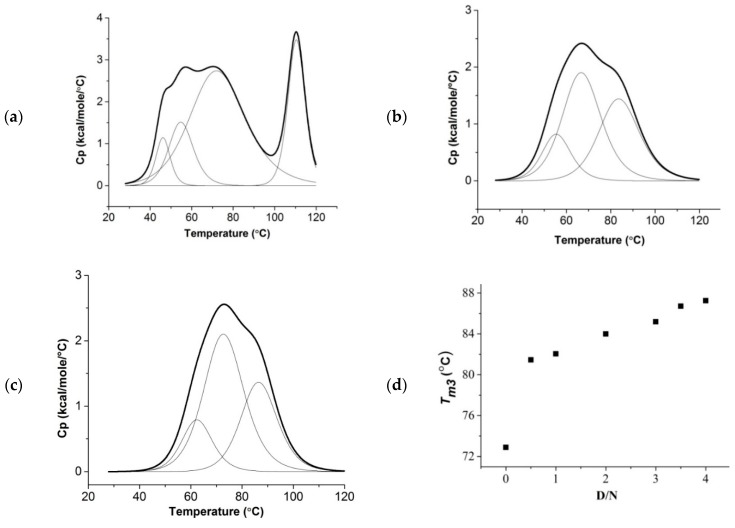
Differential scanning calorimetry (DSC) thermograms showing excess heat capacity as a function of temperature; (**a**) 50 µM of free [d-(TTGGGGT)]_4_; daunomycin-[d-(TTGGGGT)]_4_ complex at (**b**) D/N = 2.0 and (**c**) D/N = 3.5. All samples were prepared in phosphate buffer (KBPES) (pH 7.0) containing 100 mM of KCl. (**d**) The plot of melting temperature T_m3_ obtained from DSC experiments as a function of D/N ratio.

We measured the melting temperature of a folded→unfolded [d-(TTGGGGT)]_4_ G-quadruplex in free and bound form at D/N = 0.0, 0.5, 1.0, 2.0, 3.0, 3.5, and 4.0 independently using a differential scanning calorimeter (Figure 9a–c or Figure S8a–d). The thermogram of 50 μM of free [d-(TTGGGGT)]_4_ (single-strand concentration: 200 μM) fitted in a multiple-state model of deconvulation with melting transitions T_m1_ = 46.2 ± 0.4 °C, T_m2_ = 54.9 ± 0.7 °C, T_m3_ = 72.9 ± 0.6 °C, and T_m4_ = 110.5 ± 0.1 °C [39]. This in general indicates that the species may be involved in equilibria consisting of either a single-folded quadruplex to random coil transition through an intermediate, or two independent species following separate pathways not involving intermediates to the same unfolded state [48]. In the present case, the stepwise disappearance of imino protons with temperature (Figure 8) indicates the existence of several intermediates. In free DNA, G3NH and G6NH show melting around 65 °C (±5 °C), which may correspond to T_m3_ = 72.9 °C, as observed in DSC experiments. G4NH and G5NH form the core of the G tetrad, and show a melting temperature well beyond 65 °C in NMR experiments, which could be referred to as T_m4_ = 110.5 °C in DSC experiments (as T_m3_ = 72.9 °C appears to be less probable). The observed lower melting temperatures, T_m1_ = 46.2 °C and T_m2_ = 54.9 °C, may correspond to the melting transitions of other regions of [d-(TTGGGGT)]_4_ e.g., T1/T2/T7 nucleotide residues. This may be evidenced from the imino protons of thymine not being observed, since they were in fast exchange with solvent [22], as mentioned earlier. The NH melting transitions thus give insight into the stepwise melting transitions at an atomic level, which cannot be deduced from DSC experiments. In all of the complexes at D/N = 1.0–4.0 (Figure 9b,c or Figure S8a–d, Table S5), only three melting transitions were observed; the expected fourth transition is apparently beyond the range of the temperature measurements (i.e., T_m4_ > 120 °C). At D/N = 4.0, T_m1_ = 60.8 ± 1.6 °C, T_m2_ = 72.6 ± 0.4 °C, and T_m3_ = 87.3 ± 0.5 °C, and from the comparison of DSC thermograms (Figure 9a–c or Figure S8a–d) with NH melting profiles (Figure 8), it may be inferred that T_m3_ = 87.3 °C refers to the disappearance of G6NH, while the T_m4_ values for G4NH and G5NH are well beyond 120 °C. Thus, the stabilization of G6NH and G4NH/G5NH are 14.4 + 1.1 °C and >10 °C, respectively. The extent of stabilization ΔT_m_ increases with the D/N ratio (Figure 9d), which is likely to happen if both sites are not occupied at D/N = 2.0, so that binding continues as D/N approaches 4.0.

Thus, the binding of daunomycin to [d-(TTGGGGT)]_4_ leads to the thermal stabilization of the DNA quadruplex. The observed ΔT_m_ ~15 °C is significantly higher than the values at 0 °C and 5 °C that have been reported in the literature for 21-mer human telomeric DNA in Na^+^ [31] and K^+^ [32] rich aqueous solutions, respectively. The external binding of one molecule of daunomycin to 22-mer human telomeric DNA in K^+^-rich aqueous solutions yielded ΔT_m_ at ~11 °C [33]. Apparently, in the present investigations, the binding of daunomycin at two sites is stabilizing pure parallel tetramolecular [d-(TTGGGGT)]_4_ to a greater extent (ΔT_m_ ~15 °C) than unimolecular 21-mer/22-mer human telomeric DNA having a 3 + 1 hybrid structure with loops.

### 2.6. Groove Binding Mode

Daunomycin binds to both duplex and quadruplex DNA; however, it has a greater affinity for duplex DNA, although the mode of binding is totally different in the two types of DNA. MD simulations show that end-stacking with a terminal G-tetrad and the groove-binding mode are energetically equally favorable, while intercalation is the preferred binding mode over groove-binding in case of duplex DNA [25], as also reported for X-ray crystallographic [47] and NMR [42,45] structures of complexes of daunomycin–duplex DNA. The present NMR investigations confirm groove-binding and end-stacking in G-quadruplex DNA in contrast to the classical intercalation mode of binding. This may be attributed to the high stability of G-quartets and the consequently large amount of energy required to unstack them as compared to the classical intercalation of the daunomycin chromophore in d-CpG and d-TpG steps upon binding to [d-(CGATCG)]_2_, [d-(TGATCA)]_2_, etc. [42,45,47]. Groove-binding is a novel mode, since the chemical nature of the grooves in the G-quadruplex is quite different from that of duplex DNA. A large negative electrostatic field exists in the center of G-quartets as compared to the major groove of a G–C base pair in duplex DNA. The structure of distamycin [d-(TGGGGT)]_4_ [19] showed that all of the grooves were practically the same, and the ligand could easily bind at two diametrically opposite grooves. This feature in G-quadruplex DNA can provide selectivity over duplex DNA, which is essential to translate G-quadruplex ligands into successful drugs and also reduce the cell toxicity caused by presently used daunomycin/doxorubicin in chemotherapy. That daunomycin targets the pathway of telomere maintenance by telomerase paves the way to structure-based design that can produce a de novo anthracycline that acts as a more potent telomerase inhibitor. The present investigations of interactions at the molecular level by NMR contribute to the understanding of non-selective drugs such as daunomycin, which binds to both duplex and quadruplex DNA. Anthracyclines offer opportunities to have many derivatives/analogues by different chemical modifications. For instance, our studies show the involvement of the 5′CH_3_ group of daunosamine in binding. It will be worth investigating if epimer forms of sugar, or two or more conjugated sugar rings, could show an enhanced insertion in grooves and better selectivity. Also, substitution on the aromatic ring could affect binding affinity.

## 3. Materials and Methods

Daunomycin, desalted oligonucleotide sequence d-(TTGGGGT), trimethylsilyl propionic acid (TSP), and deuterated water (D_2_O) were purchased from Sigma Aldrich Chem. Co. (St Louis, MO USA), and were used directly. Other chemicals for buffer preparations such as Ethylene Diamine Tetra Acetic acid (EDTA), potassium chloride, K_2_HPO_4_, etc. were purchased from Merck Chemicals (Kenilworth, NJ, USA).

### 3.1. Surface Plasmon Resonance

Surface plasmon resonance experiments were done on a Biacore T200 instrument optical biosensor system (GE Healthcare, Chicago, IL, USA). DNA samples were immobilized onto a streptavidin-derivatized sensor chip, BIACORE SA (GE Healthcare Life Sciences, Little Chalfont, Buckinghamshire, UK), on flow cell 2. HEPES buffer (0.01 M of HEPES, 3 mM of EDTA, 0.005% P20 surfactant) containing 100 mM of KCl at pH 7.4 was passed over flow cell 1 having no immobilized DNA and used as reference. Drug solutions (3.75–960 µM) prepared in HEPES buffer containing 100 mM of KCl were passed over flow cell 2, having immobilized quadruplex [d-(TTGGGGT)]_4_ with a flow rate of 30 µL/min at 25 °C until steady-state response was reached. The drug solutions were then replaced by regeneration buffer (1 mM of NaCl and 50 µM of NaOH), causing the dissociation of the complex. The response from the reference cells i.e., flow cell 1, was subtracted from corresponding flow cell 2 to get the response from the bound ligand. The results were fitted and analyzed using the Biacore T200 evaluation software that was available with the instrument to get the binding constant (*K_b_*).

### 3.2. Job Plot Using Fluorescence

A continuous variation analysis procedure i.e., Job Plot, was used to establish the binding stoichiometry of the daunomycin [d-(TTGGGGT)]_4_ complex using fluorescence spectroscopy. Two sets of samples, that is, free daunomycin and bound daunomycin in a daunomycin–DNA complex, were prepared separately. In a set of daunomycin–DNA complexes, the total concentration of DNA and daunomycin was kept constant at 7 μM, while the relative mole fraction of daunomycin and DNA was varied. In the other set of free daunomycin, the concentration of daunomycin in a sample was kept identical to that of the corresponding sample of the daunomycin–DNA complex. The fluorescence emission was measured at λ_em_ = 592 nm using λ_ex_ = 480 nm at a constant temperature of 25 °C. The difference in the fluorescence intensity of free (F_0_) and bound (F) daunomycin (i.e., ΔF = F_0_ − F) corresponding to each sample in two sets was plotted as a function of the mole fraction of daunomycin. The inflection point indicates the change in slope, which gives the stoichiometry of the daunomycin bound to the G-quadruplex according to the following equation:

(1) $$\;n=\frac{\chi_{\mathrm{daunomycin}}}{1-{\;\chi }_{\mathrm{daunomycin}}}\;$$

where $\chi_{\mathrm{daunomycin}}$ is the mole fraction of daunomycin at the point of inflection, and *n* is the stoichiometry of the complex.

### 3.3. Circular Dichroism

Circular dichroism experiments were performed using a model Chirascan (Applied Photophysics, Leatherhead, UK) spectropolarimeter equipped with a temperature-controlled cell holder using a quartz cuvette of 10-mm path length. Before igniting the xenon lamp and throughout the course of the experiments, the instrument was continuously purged with pure nitrogen gas using a nitrogen generator (make SAS F-DGS, Every, France model HPNG10/1). The CD spectra were recorded at 25 °C using a 10-mm quartz cuvette in the range of 200–600 nm, bandwidth = 1 nm, step size = 0.5 nm, and time per point = 0.25 s. During titrations, the concentration of [d-(TTGGGGT)]_4_ was kept constant as 15.0 μM, with daunomycin added progressively to obtain various D/N ratios in one set of experiments. In another set of experiments, daunomycin concentration was kept constant at 400 μM, with DNA added stepwise to it to obtain various D/N ratios at 25 °C. The final spectra were averaged over two scans, then baseline was subtracted and smoothed by using the Savitzky–Golay algorithm provided by Chirascan software 4.5.1848.0 (Applied Photophysics, Leatherhead, UK).

### 3.4. Nuclear Magnetic Resonance

First, 1.16 mM of G-quadruplex DNA [d-(TTGGGGT)]_4_ was prepared by heating DNA sequence d-(TTGGGGT) in 10 mM of KBPES buffer (in 90% H_2_O and 10% D_2_O solvent) containing 10 mM of K_2_HPO_4_, 1 mM of EDTA, and 100 mM of KCl (pH 7.0) at 90 °C for 5 min, and kept overnight at room temperature. Drug stock was prepared in water directly, and different drug (D) and nucleic acid (N) ratios, D/N, were obtained from the titration of the drug accordingly. ^1^H and ^31^P resonances were calibrated using 0.1 µL of 0.1 M of trimethylsilyl propionic acid (TSP) as the standard internal reference, and 85% phosphoric acid as the external reference, respectively. All of the NMR spectra were recorded in a 500-MHz Bruker Avance NMR spectrometer equipped with a TXI (triple inverse) probe, BBO (Broad Band Observed) probe, and BVT (Variable Temperature Unit) at Central NMR Facility, IIT Roorkee, India. One-dimensional (1D) ^1^H spectra were recorded with the zgpr pulse program from Bruker library parameters for achieving water suppression with data points (TD) = 64 K, number of scans (NS) = 64–128, pulse width (P1) = 11.49–14.9 µs, spectral width (SW) = 20 ppm, and relaxation delay (D1) = 2.0 s at different temperatures. 1D ^31^P spectra were recorded at different temperatures by pulse program zgpg30 with TD = 64 K, NS = 64–128, P1 = 13.60–14.9 µs, SW = 10 ppm, and D1 = 2.0 s. Thermal melting studies were performed by acquiring the 1D ^1^H NMR spectra of free DNA and daunomycin [d-(TTGGGGT)]_4_ complexes at D/N ratios = 1.0–4.0 in the range of 25 °C to 80 °C. All of the samples were incubated for 10 min at respective temperatures before performing any experiment. Two-dimensional (2D) ^1^H-^1^H NOESY experiments were done using the pulse program noesyphpr with States-TPPI mode in the TXI probe at 25 °C along with mixing times (τ_m_) = 400 ms, 250 ms, and 200 ms, NS = 64, data size = 256 (t_1_) × 2048 (t_2_), P1 = 11.49–14.9 µs, SW = 20 ppm on both F1 and F2 dimensions, and D1= 1.9 s at D/N = 1.0, 2.0, 3.0, and 4.0. 2D ^13^C-^1^H Heteronuclear Single Quantum Coherence (HSQC) experiments were carried out with a TXI probe to find out the proton and carbon correlation of free daunomycin, free DNA, and daunomycin [d-(TTGGGGT)]_4_ complexes (i.e., D/N = 1.0, 2.0, 3.0, and 4.0) at 25 °C using pulse program hsqcetgpsi with NS = 64, data size = 256 (t_1_) × 2048 (t_2_), SW = 200 ppm in the F1 dimension, and 20 ppm in the F2 dimension and D1 = 1.9 s. 2D ^1^H-^31^P Heteronuclear Multiple Bond Correlation (HMBC) experiments were recorded with a BBO probe using pulse program hmbcgpndqf (gradient) at 25 °C with NS = 64, data size = 256 (t_1_) × 2048 (t_2_), SW = 10 ppm along the F1 dimension and 20 ppm along the F2 dimension, and D1 = 1.9 s at D/N = 1.0, 2.0, 3.0, and 4.0. Interproton distances were calculated using the isolated spin pair approximation (ISPA) method from the 2D ^1^H-^1^H NOESY spectra of complex recorded at 25 °C using τ_m_ = 250 ms, as it was within the linear approximation range. The integration of well-separated cross-peak volumes was done using SPARKY software 3.114, and subsequently, distances were calculated using thymine H6-CH_3_ as a reference distance (2.95 Å), assuming the NOE cross-peak intensity to be inversely proportional to the sixth power of distance. A range of ±0.5 Å was used as the distance restraint to avoid errors during integration. Diffusion Ordered SpectroscopY (DOSY) spectra was acquired for free daunomycin, free quadruplex DNA [d(TTGGGGT)]_4_, and daunomycin-[d(TTGGGGT)]_4_ complex using the following acquisition parameters: pulse program = ledbpgppr2s, data points = 16 K, spectral width = 20.0 ppm in the F2 direction and 16 free induction decays in the F1 direction with number of scans (NS) = 8. During the experiment, the diffusion time (Δ or D20) = 50 ms and diffusion gradient length (δ or P30) = 2.2 ms were kept constant, while the gradient range varied from 5 G/cm to 95 G/cm in 16 steps with a linear ramp at 25 °C. The gradient shape SMSQ 10 and 100 were used with eddy current D21 = 5 ms. The diffusion coefficients were measured from the DOSY experiment using the relation between translational self-diffusion and the measurable NMR parameters, as given by the following equation:

I/I_0_ = −exp [D_t_γ_H_^2^δ^2^G_z_^2^ (Δ − δ/3)] (2)

where I is the measured peak intensity (or volume), I_0_ is the maximum peak intensity, D_t_ is the translational diffusion constant (m^2^/s), γ_H_ is the gyromagnetic ratio of a proton (2.67 × 10^4^ G^−1^ s^−1^), δ is the duration of the gradient, Δ is the time between gradients, and G_z_ is the strength of the gradient (in G/cm). Data was plotted as −ln (I/I_0_) versus γ_H_^2^δ^2^G_z_^2^ (Δ − δ/3), and D_t_ was calculated from the slope of the plot. The diffusion coefficient (D_t_) was obtained from the slope of the linear graph of −ln (I/I_0_) versus γ_H_^2^δ^2^G_z_^2^ (Δ − δ/3) using diffusion decay curve fitting carried out with the SimFit algorithm in Topspin 3.5.

To estimate the binding affinity of the drug, the NMR titration data of the drug–DNA complex was used. We tried to fit the data in non-linear curve fitting regression analysis by giving the user-defined equation in OriginPro 2018 [49].

(3) $$\;\delta_{b}-\delta_{f}=\frac{k_{0}-k_{1}}{2}\left\{\left(C_{t}+C_{N_{t}}+\frac{1}{K_{b}}\right)-\sqrt{{\left(C_{t}+C_{N_{t}}+\frac{1}{K_{b}}\right)}^{2}-4C_{t}}C_{N_{t}}\right\}\;$$

where ***δ_f_*** and ***δ_b_*** are the chemical shift (ppm) values of the free [d(TTGGGGT)]_4_ and daunomycin [d(TTGGGGT)]_4_ complexes, respectively, ***k*_0_** and ***k*_1_** are the proportionality constants, ***C_t_*** and C***_Nt_*** are the total concentrations of drug and nucleotide respectively, and ***K_b_*** is the binding affinity.

### 3.5. Restrained Molecular Dynamics Simulations

The restrained molecular dynamics (rMD) simulations were done on a Silicon Graphics Fuel workstation enabled with INSIGHT II (version 2005, Accelrys Inc., San Diego, CA, USA) and the DISCOVER (version 2005, Accelrys Inc., San Diego, CA, USA) software program. The initial structure of the G-quadruplex DNA [d-(TTGGGGT)]_4_ was obtained from PDB ID:139D, and was used further by replacing aromatic bases in the BIOPOLYMER module (version 2005, Accelrys Inc., San Diego, CA, USA) for maintaining the potential setup. The initial structure of daunomycin was built using restraints obtained from the NOE cross-peaks builder module of INSIGHT II (version 2005). Distances were categorized as strong (s) = 2.0–3.0 Å, medium (m) = 3.0–4.0 Å, and weak (w) = 4.0–5.0 Å. The upper and lower bounds of distances were set as 0.5 Å for intermolecular restraints. A force constant of 10.0 kcal mol^−1^ Å^−2^ was used for each NOE contact. Daunomycin was placed manually at the position and distance obtained from the NOE cross-peaks and minimized using 1000 steps of steepest descent and conjugate gradient using a constant valence force field (CVFF) in DISCOVER software. Simulated annealing restrained molecular dynamics protocol was used as a tool to search for conformations that fit into the experimental NOE data and enable the visualization of 3D structural models. The molecule was heated to 800 K in steps of 100 K, so that the chances of a molecule being trapped in local minima was lost, and it reached global minima instead. The simulations were carried out for 1000 iterations, each with a time step of 1 fs at 800 K. The molecule was annealed from 800 K to 300 K with 100 K steps using MD runs of 1000 iterations of 1 fs each; thus, there was a total of 6 ps. In the end, all of the structures were minimized via 1000 steps of Steepest Descent until a predefined convergence limit of root mean square derivative of <0.001 Kcal mol^−1^ Å^−1^ was reached. The total of the 10 lowest structures was chosen for RMSD analysis out of 100 structures saved at regular intervals of 10 fs during the final step of molecular dynamics (1 ps).

### 3.6. Differential Scanning Calorimetry

Excess heat capacity as a function of temperature was measured to obtain thermal transitions of free [d-(TTGGGGT)]_4_ and its complexes with daunomycin at D/N ratios = 0.5, 1.0, 2.0, 3.0, 3.5, and 4.0 using a MicroCal VP-DSC instrument (MicroCal, Northampton, MA, USA). All of the samples were prepared in 10 mM of KBPES buffer containing 10 mM of K_2_HPO_4_, 1 mM of EDTA, and 100 mM of KCl (pH 7.0). All of the samples were properly degassed before the experiment. Each sample was scanned from 25 °C to 120 °C with a scan rate of 60 °C/h at approximately constant pressure ~33 psi. The buffer was scanned repeatedly under similar conditions as that of the sample until a proper baseline was obtained. Then, 50 µM of free [d-(TTGGGGT)]_4_ (corresponding to a single-strand concentration of d-(TTGGGGT) as 200 µM) was scanned to obtain the melting profile of free DNA. Each set of complexes with increasing daunomycin concentrations and a fixed [d-(TTGGGGT)]_4_ concentration of 50 µM at D/N ratios = 0.5, 1.0, 2.0, 3.0 and 4.0 were scanned to obtain the melting profiles of the bound forms. Each scan was subtracted from the buffer baseline. The thermograms were then deconvulated and fitted in three-state or four-state models of unbound quadruplex DNA and its complexes using the Origin 7.1 software (OriginLab Corp., Northampton, MA, USA) that was provided with the instrument.

## 4. Conclusions

The present study focuses on the binding of daunomycin to parallel-stranded tetramolecular [d-(TTGGGGT)]_4_ quadruplex DNA comprising telomeric DNA sequence from *Tetrahymena thermophilia*. Direct real-time binding experiments show specific interaction between two molecules. The daunomycin-[d-(TTGGGGT)]_4_ complex undergoes diffusion as a stable complex; complexes having stoichiometry of 1:1 and 2:1 coexist. The overall conformation of [d-(TTGGGGT)]_4_ is not significantly altered on interaction, and the daunomycin dimers present in free daunomycin are disrupted on binding to DNA. The NMR experiments confirm that a specific interaction between two molecules occurs. The presence of sequential NOEs along the sequence of DNA in ^1^H-^1^H NOESY spectra and the absence of large downfield shifts in ^31^P NMR studies give a clear proof of absence of classical intercalation to permit the stacking of the aromatic chromophore of daunomycin between G-quartets of DNA. Two binding sites, G6-T7 and T1-T2, are evident from an analysis of chemical shifts, line broadening, and 22 short interproton NOE contacts. Ring A protons—1H, 2H, 3H, 4OCH_3_—and daunosamine sugar protons—5′H, 5′CH_3_—are involved in interaction with DNA. End stacking with terminal G-tetrad G6 and groove-binding close to T1-T2 bases are found to exist. The binding leads to substantial thermal stabilization of [d-(TTGGGGT)]_4_ by Δ*T_m_* ~15 °C, which may result from an enhanced stacking of the G6 quartet with daunomycin chromophore at the G6pT7 site, as well as the presence of hydrogen bond and specific van der Waals contacts at grooves. The findings have implications in the structure-based designing of anthracycline drugs as potent telomerase inhibitors.

## Figures and Tables

**Table 1:** ^1^H Chemical shift (ppm) of protons in daunomycin [d-(TTGGGGT)]_4_ complex (δ_b_) at D/N = 4.0 in KBPES buffer containing 100 mM of KCl (90% H_2_O + 10% D_2_O solvent) at 25 °C. Δδ refers to a change in chemical shift due to binding. −ve Δδ indicates upfield shift.

Residues	T1		T2		G3		G4	
Protons	δ_b_	∆δ	δ_b_	∆δ	δ_b_	∆δ	δ_b_	∆δ
H8/H6	7.57	−0.02	7.54	0.00	8.08	−0.06	7.74	−0.06
H1′	6.15	0.01	6.05	0.02	6.03	−0.04	6.02	−0.04
CH_3_	1.60	−0.05	1.51	−0.02	-	-	-	-
NH_2_^b^	7.57	−0.02	7.54	0.00	9.73	−0.11	9.05	−0.12
NH_2_^nb^	-	-	-	-	6.14	−0.16	6.06	−0.14
NH	-	-	-	-	11.36	−0.15	10.97	−0.11
Residues	G5		G6		T7			
Protons	δ_b_	∆δ	δ_b_	∆δ	δ_b_	∆δ		
H8/H6	7.74	−0.10	7.69	−0.02	7.64	0.30		
H1′	6.02	−0.06	6.27	0.03	6.20	0.15		
CH_3_	-	-	-	-	1.84	0.21		
NH_2_^b^	9.05	−0.12	7.47	0.01	7.64	0.30		
NH_2_^nb^	7.75	−0.07	6.77	0.01	-	-		
NH	10.82	−0.12	10.55	−0.34	-	-		

**Table 2:** ^1^H Chemical shift (ppm) of daunomycin protons in free daunomycin (δ_f_) and daunomycin-[d-(TTGGGGT)]_4_ complex (δ_b_) at D/N = 4.0 in KBPES buffer containing 100 mM of KCl (90% H_2_O + 10% D_2_O solvent) at 25 °C. Change in chemical shift of daunomycin protons due to binding, Δδ = δ_b_ − δ_f_. −ve Δδ indicates upfield shift.

Protons	δ_f_	δ_b_	Δδ	Protons	δ_f_	δ_b_	Δδ
Ring D				Daunosamine sugar			
2H	7.71	7.20	−0.51	1′H	5.49	5.11	−0.38
1H	7.53	7.03	−0.50	2′eq	1.99	1.81	−0.18
3H	7.43	6.93	−0.50	2′ax	1.99	1.81	−0.18
4OCH_3_	3.94	3.60	−0.34	3′H	3.70	3.53	−0.17
Ring A				4′H	3.83	3.68	−0.15
7H	4.82	4.55	−0.27	5′H	4.27	4.05	−0.22
8eq	2.23	1.91	−0.32	5′CH_3_	1.30	1.05	−0.25
8ax	2.13	1.91	−0.22				
9COCH_3_	2.45	2.28	−0.17				
10eq	2.94	2.58	−0.36				
10ax	2.70	2.53	−0.17				

**Table 3:** Interproton distances (Å) between daunomycin and [d-(TTGGGGT)]_4_ obtained from NOE cross-peaks at D/N = 2.0, τ_m_ = 250 ms, at 25 °C and corresponding distances (Å) in rMD model of daunomycin-[d-(TTGGGGT)]_4_ complex. nd: not determined.

S. No.	Intermolecular NOE Correlations	Interproton Distances		S. No.	Intermolecular NOE Correlations	Interproton Distances	
		NOESY at D/N = 2.0	rMD Model			NOESY at D/N = 2.0	rMD Model
**1**	3H-T2CH_3_	4.22	3.44	12	4OCH_3_-T7H6	4.19	4.15
**2**	1H-T2CH_3_	4.06	3.96	13	1H-T1CH_3_	3–4	5.14
**3**	4OCH_3_-G6H1′	3.99	4.57	14	2H-G6H2′′	nd	nd
**4**	4OCH_3_-G6H8	4.20	4.83	15	1H-G6H2′′	nd	nd
**5**	4OCH_3_-G6NH	4–5	4.31	16	3H-G6H2′′	nd	nd
**6**	2H-G6NH	3–4	4.03	17	1H-G6H2′	nd	nd
**7**	1H-G6NH	3.0	5.94	18	4OCH_3_-G6NH_2_^b^	4.16	4.75
**8**	3H-G6NH	3–4	2.79	19	5′CH_3_-G6H8	nd	nd
**9**	3H-T7CH_3_	4.79	4.24	20	5′CH_3_-G6H1′	nd	nd
**10**	1H-T7CH_3_	3.11	3.02	21	5′CH_3_-G6H2′′	nd	nd

**Table 4:** Structural data and final energy terms of daunomycin-[d-(TTGGGGT)]_4_ complex.

Experimental Restraints	
Intramolecular	
Daunomycin–Daunomycin	11
DNA–DNA	138
Intermolecular	
Daunomycin–DNA	13
Total restraints	151
Average Root Mean Square Deviation (RMSD) in Å	0.35
Restraint Violations (distances > 0.3 Å)	5
Consistent Valence Force Field (CVFF) energy of minimized structures (kcal/mole)	
Total	−1641.98
Torsional	240.33
Electrostatic	−3163.33
Van der Waals	2527.77

## Supplementary Materials

The following are available online. Table S1 (a): 1H Chemical shift (ppm) of DNA protons in daunomycin-[d-(TTGGGGT)]_4_ complex (δb) at various Daunomycin (D)/Nucleic acid (N) ratios, D/N, in KBPES buffer containing 100 mM KCl (90% H2O+10% D2O) at 25 °C. Δδ refers to change in chemical shift due to binding, Table S1 (b): 1H Chemical shift (ppm) of DNA protons in daunomycin-[d-(TTGGGGT)]_4_ complex (δb) at various D/N ratios in KBPES buffer containing 100 mM KCl (90% H_2_O + 10% D_2_O) at 25 °C. Δδ refers to change in chemical shift due to binding, Table S1 (c): 1H Chemical shift (ppm) of daunomycin protons in free daunomycin (δf) and in daunomycin-[d-(TTGGGGT)]_4_ complex (δb) at various D/N ratios in KBPES buffer containing 100 mM KCl (90% H_2_O + 10% D_2_O) at 25 °C. Δδ = δb − δf, nd: not determined, Table S2 (a) 1H Chemical shift (ppm) of methyl and imino protons in free [d-(TTGGGGT)]_4_ (δf) and daunomycin-[d-(TTGGGGT)]_4_ complex (δb) at various D/N ratios in KBPES buffer containing 100 mM KCl (90% H_2_O + 10% D_2_O) at 25 °C. Δδ = δb − δf, Table S2 (b) 1H Chemical shift (ppm) of base protons in free [d-(TTGGGGT)]_4_ (δf) and daunomycin-[d-(TTGGGGT)]_4_ complex (δb) at various D/N ratios in KBPES buffer containing 100 mM KCl (90% H_2_O + 10% D_2_O) at 25 °C. Δδ = δb − δf, Table S2 (c): 1H Chemical shift (ppm) of H1′ sugar protons in free [d-(TTGGGGT)]_4_ (δf) and daunomycin-[d-(TTGGGGT)]_4_ complex (δb) at various D/N ratios in KBPES buffer containing 100 mM KCl (90% H_2_O + 10% D_2_O) at 25 °C. Δδ = δb − δf, Table S2 (d): 1H Chemical shift (ppm) of daunomycin protons in free state (δf) and in daunomycin-[d-(TTGGGGT)]_4_ complex (δb) at various D/N ratios in KBPES buffer containing 100 mM KCl (90% H_2_O + 10% D_2_O) at 25 °C. Δδ = δb − δf, Table S3: Intra molecular distances (Å) and relative intensities of intra molecular NOE cross peaks of daunomycin protons in daunomycin-[d-(TTGGGGT)]_4_ complex at D/N = 1.0, 2.0, 3.0 and 4.0 at τm = 250 ms at 25 °C. nd: not determined, Table S4: Chemical shift (ppm) of phosphorus (31P) resonances in free [d-(TTGGGGT)]_4_ (δf) and daunomycin-[d-(TTGGGGT)]_4_ complex (δb) at various D/N ratios in KBPES buffer containing 100 mM KCl (90% H_2_O + 10% D_2_O) at 25 °C. Δδ = δb − δf, Table S5: Melting temperature (Tm) of [d-(TTGGGGT)]_4_ in free state and in daunomycin-[d-(TTGGGGT)]_4_ complex at different D/N ratios obtained from Differential Scanning Calorimetry experiments. Change in melting temperature (ΔTm) due to binding is also shown. nd: not determined, Figure S1 (a,b): 1D 1H NMR spectra of free [d-(TTGGGGT)]_4_ in KBPES buffer containing 100 mM KCl (90% H_2_O + 10% D_2_O) at 25 °C. Expansion of 1H-1H 2D NOESY spectra of free [d-(TTGGGGT)]_4_ showing sequential connectivity (black arrows), Figure S2: 1D 1H NMR spectrum of free daunomycin in water (90% H_2_O + 10% D_2_O) at 25 °C, Figure S3 (a,b): 1D 1H NMR spectrum of daunomycin-DNA [d-(TTGGGGT)]_4_ complex at D/N = 2.0 in KBPES buffer containing 100 mM KCl (90% H_2_O + 10% D_2_O) at 25 °C. Daunomycin protons are represented by (#). Expansion of 1H-1H 2D NOESY spectra of daunomycin-DNA [d-(TTGGGGT)]_4_ complex at D/N = 2.0 showing sequential connectivity (black arrows) between (c) imino protons (d) adjacent imino and base protons (e) base-sugar (H1′) protons (f) base-sugar (H2′/2′′) protons at τm = 250 ms in KBPES buffer (90% H_2_O + 10% D_2_O) at 25 °C, Figure S4 (a,d) Expansion of specific region of 2D 1H-13C HSQC spectrum showing overlay of free daunomcin (red), free [d-(TTGGGGT)]_4_ (green) and daunomycin-[d-(TTGGGGT)]_4_ complex at D/N = 2.0 (blue) in KBPES buffer (90% H_2_O + 10% D_2_O) at 25 °C. 1D 1H NMR spectrum at the top of HSQC spectra is daunomycin-DNA [d-(TTGGGGT)]_4_ complex at D/N = 2.0, Figure S5: Nonlinear fitted curve (red) for simultaneous binding at two different sites in daunomycin-[d(TTGGGGT)]_4_ complex of protons, Figure S6: 2D 1H-31P HMBC spectra, Figure S7: 2D 1H-1H NOESY spectra of daunomycin-[d-(TTGGGGT)]_4_ complex at D/N = 2.0, mixing time (τm) = 250 ms at 25 °C, Figure S8: Differential Scanning Calorimetry (DSC) thermograms showing excess heat capacity as a function of temperature for daunomycin-[d-(TTGGGGT)]_4_ complex.
