# Supplementary material for: Molecular Recognition of Parallel G-quadruplex [d-(TTGGGGT)]4 Containing Tetrahymena Telomeric DNA Sequence by Anticancer Drug Daunomycin: NMR-Based Structure and Thermal Stability
Source: Molecules. 2018 Sep 5;23(9):2266. doi: 10.3390/molecules23092266 (PMC6225185; doi:10.3390/molecules23092266)
Supplement: Supplementary file 1 [file molecules-23-02266-s001.pdf]

Supplementary Information

Molecular Recognition of Parallel G-quadruplex [d-(TTGGGGT)]$_4$ containing *Tetrahymena* Telomeric Repeat by Anticancer Drug Daunomycin: NMR based Structure and Thermal Stability

Ritu Barthwal,* Zia Tariq

Department of Biotechnology, Indian Institute of Technology Roorkee, Roorkee 247667, India

*Corresponding author

Address for correspondence:
Prof. Ritu Barthwal, Ph. D
Department of Biotechnology,
Indian Institute of Technology Roorkee,
Roorkee 247667, India.
E-mail: ritubfbs@iitr.ac.in, barthwalritu.iitr@gmail.com
Fax: +91-1332-273560; Phone: +91-1332-285807

Tables

Table S1 a) [1]H Chemical shift (ppm) of DNA protons in daunomycin-[d-(TTGGGGT)]$_4$ complex (δ_b) at various Daunomycin (D)/ Nucleic acid (N) ratios, D/N, in KBPES buffer containing 100 mM KCl (90% H_2O+10% D_2O) at 25 °C. $\Delta\delta$ refers to change in chemical shift due to binding.

D/N	RESIDUES	T1		T2		G3		G4		G5		G6		T7	
	PROTONS	δ_b	$\Delta\delta$	δ_b	$\Delta\delta$	δ_b	$\Delta\delta$	δ_b	$\Delta\delta$	δ_b	$\Delta\delta$	δ_b	$\Delta\delta$	δ_b	$\Delta\delta$
1.0	H8/H6	7.57	-0.02	7.53	-0.01	8.12	-0.02	7.78	-0.02	7.79	-0.05	7.72	0.01	7.59	0.25
	H1’	6.14	0.00	6.03	0.00	6.04	-0.03	6.05	-0.01	6.07	-0.01	6.30	0.06	6.18	0.13
	H2’	2.21	0.00	2.26	-0.01	2.64	-0.02	2.65	0.01	2.71	-0.01	2.26	0.00	2.28	0.11
	H2”	2.41	-0.01	2.58	-0.02	2.94	-0.02	2.91	-0.01	2.75	-0.02	2.66	-0.02	2.65	0.00
	H3’	4.62	-0.03	4.91	0.01	5.04	-0.02	5.06	0.01	5.06	-0.03	5.08	0.04	5.03	0.07
	H4’	4.26	-0.04	4.20	0.03	4.38	-0.02	4.29	-0.04	4.54	-0.04	4.52	-0.01	4.55	0.09
	H5’	4.08	0.01	4.06	0.00	4.14	-0.01	4.28	0.00	4.36	0.00	4.23	-0.02	4.15	0.02
	H5”	3.74	-0.03	4.02	-0.01	4.10	-0.01	4.23	0.00	4.28	-0.04	4.20	-0.01	4.08	0.01
	CH_3	1.63	-0.02	1.52	-0.01	-	-	-	-	-	-	-	-	1.80	0.17
	NH_2^b	-	-	-	-	9.81	-0.03	9.10	-0.07	9.09	-0.08	7.46	0.00	-	-
	NH_2^{nb}	-		-	-	6.16	-0.14	6.13	-0.07	7.79	-0.03	6.76	0.00	-	-
	NH	-	-	-	-	11.47	-0.04	11.04	-0.04	10.87	-0.07	10.67	-0.22	-	-
2.0	H8/H6	7.57	-0.02	7.54	0.00	8.11	-0.03	7.77	-0.03	7.77	-0.07	7.72	0.01	7.64	0.30
	H1’	6.15	0.01	6.05	0.02	6.04	-0.03	6.04	-0.02	6.04	-0.04	6.31	0.07	6.21	0.16
	H2’	2.23	0.02	2.24	-0.03	2.63	-0.03	2.66	0.02	2.70	-0.02	2.31	0.05	2.30	0.13
	H2”	2.44	0.02	2.57	-0.03	2.93	-0.03	2.85	-0.07	2.85	0.08	2.68	0.00	2.65	0.00
	H3’	4.66	0.01	4.92	0.02	5.04	-0.02	5.07	0.02	5.07	-0.02	5.08	0.04	5.06	0.10
	H4’	4.25	-0.05	4.19	0.02	4.38	-0.02	4.29	-0.04	-	-	4.54	0.01	4.57	0.11
	H5’	4.07	0.00	4.06	0.00	4.15	0.00	4.28	0.00	4.36	0.00	4.24	-0.01	4.16	0.03
	H5”	3.76	-0.01	4.02	-0.01	4.11	0.00	4.23	0.00	4.28	-0.04	4.20	-0.01	4.11	0.04
	CH_3	1.61	-0.04	1.54	0.01	-	-	-	-	-	-	-	-	1.84	0.21
	NH_2^b	-	-	-	-	9.78	-0.06	9.08	-0.09	9.09	-0.08	7.47	0.01	-	-
	NH_2^{nb}	-	-	-	-	6.18	-0.12	6.10	-0.10	7.76	-0.06	6.77	0.01	-	-
	NH	-	-	-	-	11.42	-0.09	11.01	-0.07	10.84	-0.10	10.59	-0.30	-	-

* Negative sign in $\Delta\delta$ indicates upfield shift.

Table S1 b) [1]H Chemical shift (ppm) of DNA protons in daunomycin-[d-(TTGGGGT)]$_4$ complex (δ_b) at various D/N ratios in KBPES buffer containing 100 mM KCl (90% H_2O+10% D_2O) at 25 °C. $\Delta\delta$ refers to change in chemical shift due to binding.

D/N	RESIDUES	T1		T2		G3		G4		G5		G6		T7	
	PROTONS	δ_b	$\Delta\delta$	δ_b	$\Delta\delta$	δ_b	$\Delta\delta$	δ_b	$\Delta\delta$	δ_b	$\Delta\delta$	δ_b	$\Delta\delta$	δ_b	$\Delta\delta$
3.0	H8/H6	7.57	-0.02	7.54	0.00	8.09	-0.05	7.75	-0.05	7.75	-0.09	7.70	-0.01	7.64	0.30
	H1’	6.17	0.03	6.06	0.03	6.04	-0.03	6.04	-0.02	6.04	-0.04	6.29	0.05	6.21	0.16
	H2’	2.23	0.02	2.24	-0.03	2.62	-0.04	2.67	0.03	2.71	-0.01	2.36	0.10	2.31	0.14
	H2”	2.47	0.05	2.56	-0.04	2.91	-0.05	2.91	-0.01	2.76	-0.01	2.66	-0.02	2.63	-0.02
	H3’	4.66	0.01	4.92	0.02	5.04	-0.02	5.05	0.00	5.05	-0.04	5.08	0.04	5.07	0.11
	H4’	4.26	-0.04	4.17	0.00	4.37	-0.03	4.29	-0.04	-	-	4.54	0.01	4.56	0.10
	H5’	4.08	0.01	4.07	0.01	4.13	-0.02	4.28	0.00	4.36	0.00	4.25	0.00	4.15	0.02
	H5”	3.76	-0.01	4.03	0.00	4.10	-0.01	4.23	0.00	4.28	-0.04	4.21	0.00	4.10	0.03
	CH_3	1.60	-0.05	1.52	-0.01	-	-	-	-	-	-	-	-	1.83	0.20
	NH_2^b	-	-	-	-	9.76	-0.08	9.06	-0.11	9.05	-0.12	7.46	0.00	-	-
	NH_2^{nb}	-	-	-	-	6.15	-0.15	6.07	-0.13	7.75	-0.07	6.76	0.00	-	-
	NH	-	-	-	-	11.37	-0.14	10.98	-0.10	10.82	-0.12	10.55	-0.34	-	-
4.0	H8/H6	7.57	-0.02	7.54	0.00	8.08	-0.06	7.74	-0.06	7.74	-0.10	7.69	-0.02	7.64	0.30

H1’	6.15	0.01	6.05	0.02	6.03	-0.04	6.02	-0.04	6.02	-0.06	6.27	0.03	6.20	0.15
H2’	2.23	0.02	2.22	-0.05	2.60	-0.06	2.67	0.03	2.67	-0.05	2.28	0.02	2.30	0.13
H2”	2.45	0.03	2.56	-0.04	2.90	-0.06	2.91	-0.01	2.79	0.02	2.65	-0.03	2.63	-0.02
H3’	4.66	0.01	4.92	0.02	5.03	-0.03	5.05	0.00	5.05	-0.04	5.07	0.03	5.07	0.11
H4’	4.26	-0.04	4.18	0.01	4.38	-0.02	4.28	-0.05	4.58	0.00	4.54	0.01	4.56	0.10
H5’	4.07	0.00	4.07	0.01	4.14	-0.01	4.28	0.00	4.36	0.00	4.24	-0.01	4.15	0.02
H5”	3.77	0.00	4.02	-0.01	4.10	-0.01	4.23	0.00	4.28	-0.04	4.20	-0.01	4.07	0.00
CH_3	1.60	-0.05	1.51	-0.02	-	-	-	-	-	-	-	-	1.84	0.21
NH_2^b	-	-	-	-	9.73	-0.11	9.05	-0.12	9.05	-0.12	7.47	0.01	-	-
NH_2^{nb}	-	-	-	-	6.14	-0.16	6.06	-0.14	7.75	-0.07	6.77	0.01	-	-
NH	-	-	-	-	11.36	-0.15	10.97	-0.11	10.82	-0.12	10.55	-0.34	-	-

* Negative sign in Δδ denotes upfield shift.

Table S1 c) [1]H Chemical shift (ppm) of daunomycin protons in free daunomycin (δ_f) and in daunomycin-[d-(TTGGGGT)]$_4$ complex (δ_b) at various D/N ratios in KBPES buffer containing 100 mM KCl (90% H_2O+10% D_2O) at 25 °C. $\Delta\delta = \delta_b - \delta_f$, nd: not determined

Daunomycin Protons	Free Daunomycin	D/N=1.0		D/N=2.0		D/N=3.0		D/N=4.0	
	δ_f	δ_b	$\Delta\delta$	δ_b	$\Delta\delta$	δ_b	$\Delta\delta$	δ_b	$\Delta\delta$
2H	7.71	7.30	-0.41	7.26	-0.45	7.19	-0.52	7.20	-0.51
1H	7.53	7.21	-0.32	7.18	-0.35	7.09	-0.44	7.03	-0.50
3H	7.43	7.04	-0.39	7.02	-0.41	6.95	-0.48	6.93	-0.50
4OCH$_3$	3.94	3.70	-0.24	3.67	-0.27	3.62	-0.32	3.60	-0.34
1'H	5.49	nd	nd	4.92	-0.57	5.06	-0.43	5.11	-0.38
7H	4.82	nd	nd	4.57	-0.25	4.54	-0.28	4.55	-0.27
5'H	4.27	3.81	-0.46	3.91	-0.36	3.98	-0.29	4.05	-0.22
4'H	3.83	3.53	-0.3	3.60	-0.23	3.64	-0.19	3.68	-0.15
3'H	3.70	3.33	-0.37	3.42	-0.28	3.48	-0.22	3.53	-0.17
5'CH$_3$	1.30	0.74	-0.56	0.90	-0.4	1.02	-0.28	1.05	-0.25
9COCH$_3$	2.45	2.43	-0.02	2.33	-0.12	2.28	-0.17	2.28	-0.17
2'eq	1.99	1.66	-0.33	1.77	-0.22	1.78	-0.21	1.81	-0.18
2'ax	1.99	1.56	-0.43	1.73	-0.26	1.73	-0.26	1.81	-0.18
8eq	2.23	2.08	-0.15	1.87	-0.36	1.91	-0.32	1.91	-0.32
8ax	2.13	1.72	-0.41	1.81	-0.32	1.85	-0.28	1.91	-0.22
10eq	2.94	2.64	-0.3	2.59	-0.35	2.56	-0.38	2.58	-0.36
10ax	2.70	2.56	-0.14	2.54	-0.16	2.53	-0.17	2.53	-0.17

* Negative sign in $\Delta\delta$ denotes upfield shift.

Table S2 a) [1]H Chemical shift (ppm) of methyl and imino protons in free $[d\text{-}(TTGGGGT)]_4$ (δ_f) and daunomycin-$[d\text{-}(TTGGGGT)]_4$ complex (δ_b) at various D/N ratios in KBPES buffer containing 100 mM KCl (90% H_2O + 10% D_2O) at 25 °C. $\Delta\delta = \delta_b - \delta_f$

**Protons**	**CH₃**						**NH**							
	**T1**		**T2**		**T7**		**G3**		**G4**		**G5**		**G6**	
Free DNA (δ_f)	1.65		1.53		1.63		11.51		11.08		10.94		10.89	
	δ_b	$\Delta\delta$	δ_b	$\Delta\delta$	δ_b	$\Delta\delta$	δ_b	$\Delta\delta$	δ_b	$\Delta\delta$	δ_b	$\Delta\delta$	δ_b	$\Delta\delta$
D/N=0.2	1.65	0.00	1.53	0.00	1.66	0.03	11.50	-0.01	11.07	-0.01	10.93	-0.01	10.86	-0.03
D/N=0.5	1.65	0.00	1.53	0.00	1.72	0.09	11.47	-0.04	11.06	-0.02	10.90	-0.04	10.80	-0.09
D/N=0.8	1.65	0.00	1.53	0.00	1.78	0.15	11.48	-0.03	11.05	-0.03	10.88	-0.06	10.70	-0.19
D/N=1.0	1.64	-0.01	1.53	0.00	1.80	0.17	11.47	-0.04	11.04	-0.04	10.87	-0.07	10.67	-0.22
D/N=1.5	1.63	-0.02	1.52	-0.01	1.83	0.20	11.45	-0.06	11.02	-0.06	10.85	-0.09	10.62	-0.27
D/N=2.0	1.62	-0.03	1.52	-0.01	1.84	0.21	11.42	-0.09	11.01	-0.07	10.84	-0.10	10.59	-0.30
D/N=2.5	1.61	-0.04	1.52	-0.01	1.84	0.21	11.39	-0.12	11.00	-0.08	10.83	-0.11	10.58	-0.31
D/N=3.0	1.61	-0.04	1.52	-0.01	1.84	0.21	11.37	-0.14	10.98	-0.10	10.82	-0.12	10.55	-0.34
D/N=3.5	1.60	-0.05	1.52	-0.01	1.84	0.21	11.35	-0.16	10.97	-0.11	10.82	-0.12	10.55	-0.34
D/N=4.0	1.60	-0.05	1.52	-0.01	1.84	0.21	11.36	-0.15	10.97	-0.11	10.82	-0.12	10.55	-0.34

* Negative sign in $\Delta\delta$ denotes upfield shift

Table S2 b) [1]H Chemical shift (ppm) of base protons in free $[d\text{-}(TTGGGGT)]_4$ (δ_f) and daunomycin-$[d\text{-}(TTGGGGT)]_4$ complex (δ_b) at various D/N ratios in KBPES buffer containing 100 mM KCl (90% H_2O + 10% D_2O) at 25 °C. $\Delta\delta = \delta_b - \delta_f$

Protons	H8/H6													
	T1		T2		G3		G4		G5		G6		T7	
Free DNA (δ_f)	7.59		7.54		8.14		7.80		7.84		7.71		7.34	
	δ_b	$\Delta\delta$	δ_b	$\Delta\delta$	δ_b	$\Delta\delta$	δ_b	$\Delta\delta$	δ_b	$\Delta\delta$	δ_b	$\Delta\delta$	δ_b	$\Delta\delta$
D/N=0.2	7.57	-0.02	7.54	0.00	8.13	-0.01	7.80	0.00	7.83	-0.01	7.71	0.00	7.35	0.01
D/N=0.5	7.57	-0.02	7.54	0.00	8.13	-0.01	7.79	-0.01	7.82	-0.02	7.71	0.00	7.44	0.10
D/N=0.8	7.57	-0.02	7.54	0.00	8.12	-0.02	7.78	-0.02	7.8	-0.04	7.72	0.01	7.55	0.21
D/N=1.0	7.57	-0.02	7.53	-0.01	8.12	-0.02	7.78	-0.02	7.79	-0.05	7.72	0.01	7.59	0.25
D/N=1.5	7.57	-0.02	7.54	0.00	8.11	-0.03	7.78	-0.02	7.79	-0.05	7.72	0.01	7.63	0.29
D/N=2.0	7.57	-0.02	7.54	0.00	8.11	-0.03	7.77	-0.03	7.77	-0.07	7.72	0.01	7.64	0.30
D/N=2.5	7.57	-0.02	7.54	0.00	8.10	-0.04	7.76	-0.04	7.76	-0.08	7.71	0.00	7.65	0.31
D/N=3.0	7.57	-0.02	7.54	0.00	8.09	-0.05	7.75	-0.05	7.75	-0.09	7.70	-0.01	7.64	0.30
D/N=3.5	7.56	-0.03	7.54	0.00	8.07	-0.07	7.74	-0.06	7.74	-0.10	7.70	-0.01	7.64	0.30
D/N=4.0	7.57	-0.02	7.54	0.00	8.08	-0.06	7.74	-0.06	7.74	-0.10	7.69	-0.02	7.64	0.30

* Negative sign in $\Delta\delta$ denotes upfield shift.

Table S2 (c) ^{1}H Chemical shift (ppm) of H1' sugar protons in free [d-(TTGGGGT)]$_4$ (δ_f) and daunomycin-[d-(TTGGGGT)]$_4$ complex (δ_b) at various D/N ratios in KBPES buffer containing 100 mM KCl (90% H_2O + 10% D_2O) at 25 °C. $\Delta\delta = \delta_b - \delta_f$

**Protons**	**H1'**					
	**T1**		**G6**		**T7**	
Free DNA (δ_f)	6.14		6.24		6.05	
	δ_b	$\Delta\delta$	δ_b	$\Delta\delta$	δ_b	$\Delta\delta$
D/N=0.2	6.14	0.00	6.25	0.01	6.06	0.01
D/N=0.5	6.13	-0.01	6.28	0.04	6.10	0.05
D/N=0.8	6.13	-0.01	6.29	0.05	6.17	0.12
D/N=1.0	6.14	0.00	6.30	0.06	6.18	0.13
D/N=1.5	6.15	0.01	6.30	0.06	6.21	0.16
D/N=2.0	6.15	0.01	6.31	0.07	6.21	0.16
D/N=2.5	6.16	0.02	6.30	0.06	6.21	0.16
D/N=3.0	6.17	0.03	6.29	0.05	6.21	0.16
D/N=3.5	6.16	0.02	6.28	0.04	6.20	0.15
D/N=4.0	6.15	0.01	6.27	0.03	6.20	0.15

* Negative sign in $\Delta\delta$ denotes upfield shift.

Table S2 d) [1]H Chemical shift (ppm) of daunomycin protons in free state (δ_f) and in daunomycin-[d-(TTGGGGT)]$_4$ complex (δ_b) at various D/N ratios in KBPES buffer containing 100 mM KCl (90% H_2O + 10% D_2O) at 25 °C. $\Delta\delta = \delta_b - \delta_f$

**Protons**	**Daunomycin**							
	**1H**		**2H**		**3H**		**5'CH$_3$**	
Free Daunomycin (δ_f)	7.53		7.71		7.43		1.30	
	δ_b	$\Delta\delta$	δ_b	$\Delta\delta$	δ_b	$\Delta\delta$	δ_b	$\Delta\delta$
D/N=0.2	7.11	-0.42	7.20	-0.51	7.03	-0.40	0.69	-0.61
D/N=0.5	7.21	-0.32	7.30	-0.41	7.04	-0.39	0.70	-0.60
D/N=0.8	7.21	-0.32	7.30	-0.41	7.04	-0.39	0.72	-0.58
D/N=1.0	7.21	-0.32	7.30	-0.41	7.04	-0.39	0.74	-0.56
D/N=1.5	7.20	-0.33	7.29	-0.42	7.04	-0.39	0.81	-0.49
D/N=2.0	7.18	-0.35	7.26	-0.45	7.02	-0.41	0.90	-0.40
D/N=2.5	7.14	-0.39	7.22	-0.49	7.00	-0.43	0.97	-0.33
D/N=3.0	7.09	-0.44	7.19	-0.52	6.95	-0.48	1.00	-0.30
D/N=3.5	7.07	-0.46	7.17	-0.54	6.95	-0.48	1.05	-0.25
D/N=4.0	7.03	-0.50	7.16	-0.51	6.93	-0.50	1.05	-0.25

*Negative sign in $\Delta\delta$ denotes upfield shift.

Table S3 Intra molecular distances (Å) and relative intensities of intra molecular NOE cross peaks of daunomycin protons in daunomycin-[d-(TTGGGGT)]$_4$ complex at D/N = 1.0, 2.0, 3.0 and 4.0 at τ_m = 250 ms at 25 °C. nd: not determined

S.No.	Label	Intra molecular NOE correlations	Inter proton distance at D/N=2.0	Intensity of NOE cross peak at D/N=1.0	Intensity of NOE cross peak at D/N=2.0	Intensity of NOE cross peak at D/N=3.0	Intensity of NOE cross peak at D/N=4.0
1	D1	5'CH_3-5'H	2.70	s	s	s	s
2	D2	5'CH_3-4'H	2.74	s	s	s	s
3	D3	5'CH_3-3'H	3.58	w	m	w	nd
4	D4	$2'_{eq}$-4'H	2.95	m	m	m	nd
5	D5	$2'_{eq}$-3'H	2.66	s	s	s	nd
6	D6	4OCH_3-2H	2.90	s	s	s	s
7	D7	4OCH_3-1H	3-4	m	m/broad	m/broad	m/broad
8	D8	4OCH_3-3H	2.20	s	s	s	s
9	D9	5'H-4'H	2.70	nd	s	nd	nd
10	D10	5'H-3'H	3.01	nd	m	m	nd
11	D11	8_{ax}-5'H	2.72	s	s	s	nd

s - strong, m - medium, w – weak intensity; o- overlap

Table S4: Chemical shift (ppm) of phosphorus (^{31}P) resonances in free [d-(TTGGGGT)]$_4$ (δ_f) and daunomycin-[d-(TTGGGGT)]$_4$ complex (δ_b) at various D/N ratios in KBPES buffer containing 100 mM KCl (90% H_2O + 10% D_2O) at 25 °C. $\Delta\delta = \delta_b - \delta_f$

	T1pT2		T2pG3		G3pG4		G4pG5		G5pG6		G6pT7	
Free DNA (δ_f)	-0.44		-0.69		-0.78		-0.90		-0.87		-0.77	
	δ_b	$\Delta\delta$	δ_b	$\Delta\delta$	δ_b	$\Delta\delta$	δ_b	$\Delta\delta$	δ_b	$\Delta\delta$	δ_b	$\Delta\delta$
D/N=0.2	-0.47	-0.03	-0.71	-0.02	-0.79	-0.01	-0.93	-0.03	-0.90	-0.03	-0.77	0.00
D/N=0.5	-0.50	-0.06	-0.74	-0.05	-0.81	-0.03	-0.97	-0.07	-0.94	-0.07	-0.75	0.02
D/N=0.8	-0.53	-0.09	-0.77	-0.08	-0.84	-0.06	-1.01	-0.11	-0.98	-0.11	-0.73	0.04
D/N=1.0	-0.55	-0.11	-0.79	-0.10	-0.86	-0.08	-1.03	-0.13	-1.01	-0.14	-0.72	0.05
D/N=1.5	-0.58	-0.14	-0.82	-0.13	-0.89	-0.11	-1.08	-0.18	-1.07	-0.20	-0.74	0.03
D/N=2.0	-0.60	-0.16	-0.84	-0.15	-0.93	-0.15	-1.13	-0.23	-1.11	-0.24	-0.77	0.00
D/N=2.5	-0.63	-0.19	-0.86	-0.17	-0.95	-0.17	-1.17	-0.27	-1.15	-0.28	-0.80	-0.03
D/N=3.0	-0.63	-0.19	-0.84	-0.15	-0.95	-0.17	-1.18	-0.28	-1.16	-0.29	-0.80	-0.03
D/N=3.5	-0.68	-0.24	-0.89	-0.20	-1.01	-0.23	-1.25	-0.35	-1.23	-0.36	-0.87	-0.10
D/N=4.0	-0.71	-0.27	-0.92	-0.23	-1.03	-0.25	-1.28	-0.38	-1.26	-0.39	-0.90	-0.13

*Negative sign in $\Delta\delta$ denotes upfield shift.

Table S5 Melting temperature (T_m) of [d-(TTGGGGT)]$_4$ in free state and in daunomycin-[d-(TTGGGGT)]$_4$ complex at different D/N ratios obtained from Differential Scanning Calorimetry experiments. Change in melting temperature (ΔT_m) due to binding is also shown. nd: not determined

	T_{m1} (°C)		T_{m2} (°C)		T_{m3} (°C)		T_{m4} (°C)
Free DNA	46.2±0.4		54.9±0.7		72.9±0.6		110.5±0.1
Complex		ΔT_{m1}		ΔT_{m2}		ΔT_{m3}	ΔT_{m4}
D/N=0.5	51.0±0.4	4.8	66.7±0.3	11.8	81.5±0.7	8.6	nd
D/N=1.0	52.4±4.8	6.2	65.1±3.3	10.2	82.0±2.9	9.1	nd
D/N=2.0	55.7±3.3	9.5	67.1±2.3	12.2	84.0±1.3	11.1	nd
D/N=3.0	55.1±1.3	8.9	69.0±1.1	14.1	85.2±0.8	12.3	nd
D/N=3.5	62.6±1.4	16.4	73.0±0.7	18.1	86.7±0.5	13.8	nd
D/N=4.0	60.8±1.6	14.6	72.6±0.4	17.7	87.3±0.5	14.4	nd

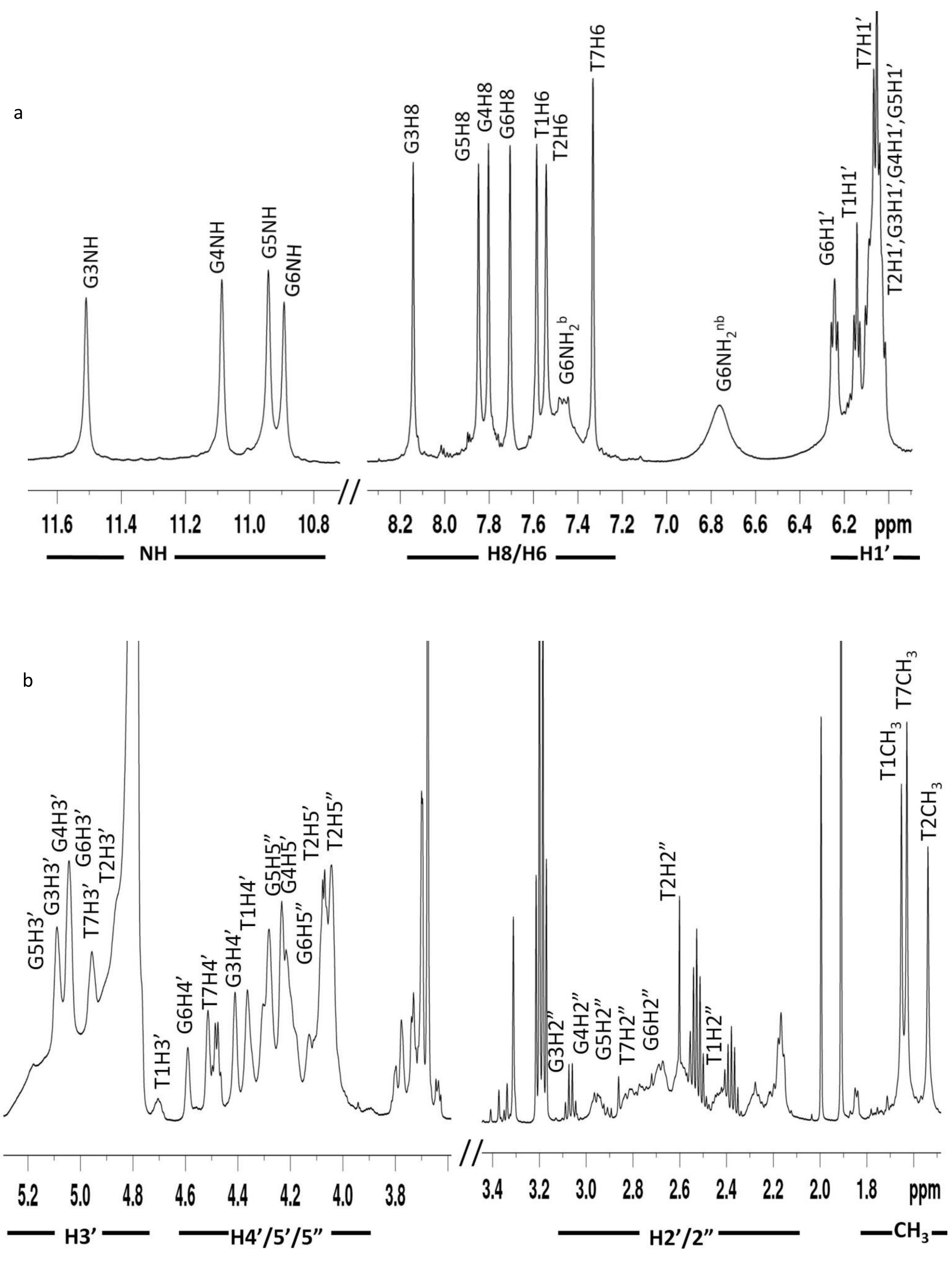
a
G3NH
G4NH
G5NH
G6NH
G3H8
G5H8
G4H8
G6H8
T1H6
T2H6
T7H6
G6NH2b
G6NH2nb
G6H1'
T1H1'
T7H1'
T2H1',G3H1',G4H1',G5H1'
11.6
11.4
11.2
11.0
10.8
8.2
8.0
7.8
7.6
7.4
7.2
7.0
6.8
6.6
6.4
6.2
ppm
NH
H8/H6
H1'
b
G5H3'
G3H3'
G4H3'
G6H3'
T7H3'
T2H3'
T1H3'
G6H4'
T7H4'
G3H4'
T1H4'
G5H5''
G4H5'
G6H5''
T2H5'
T2H5''
G3H2''
G4H2''
G5H2''
T7H2''
G6H2''
T2H2''
T1H2''
T1CH3
T7CH3
T2CH3
5.2
5.0
4.8
4.6
4.4
4.2
4.0
3.8
3.4
3.2
3.0
2.8
2.6
2.4
2.2
2.0
1.8
ppm
H3'
H4'/5'/5''
H2'/2''
CH3

c

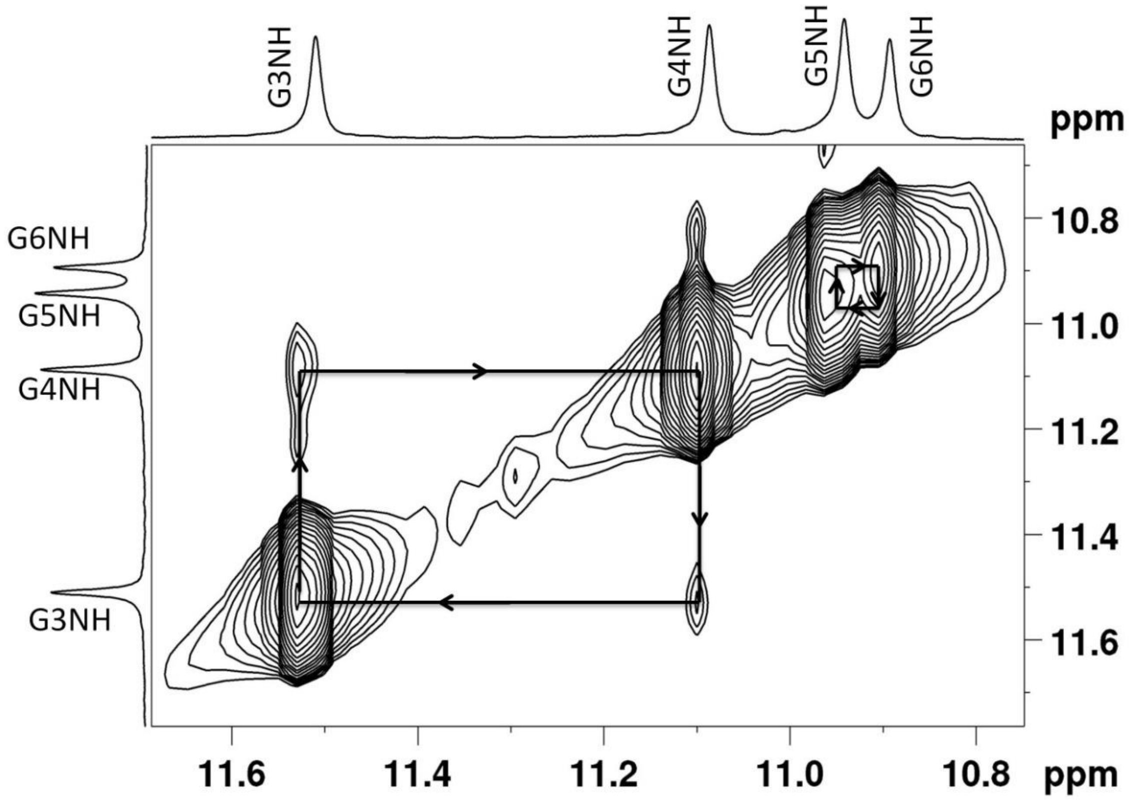
G3NH
G4NH
G5NH
G6NH
ppm
10.8
11.0
11.2
11.4
11.6
11.6
11.4
11.2
11.0
10.8
ppm

d

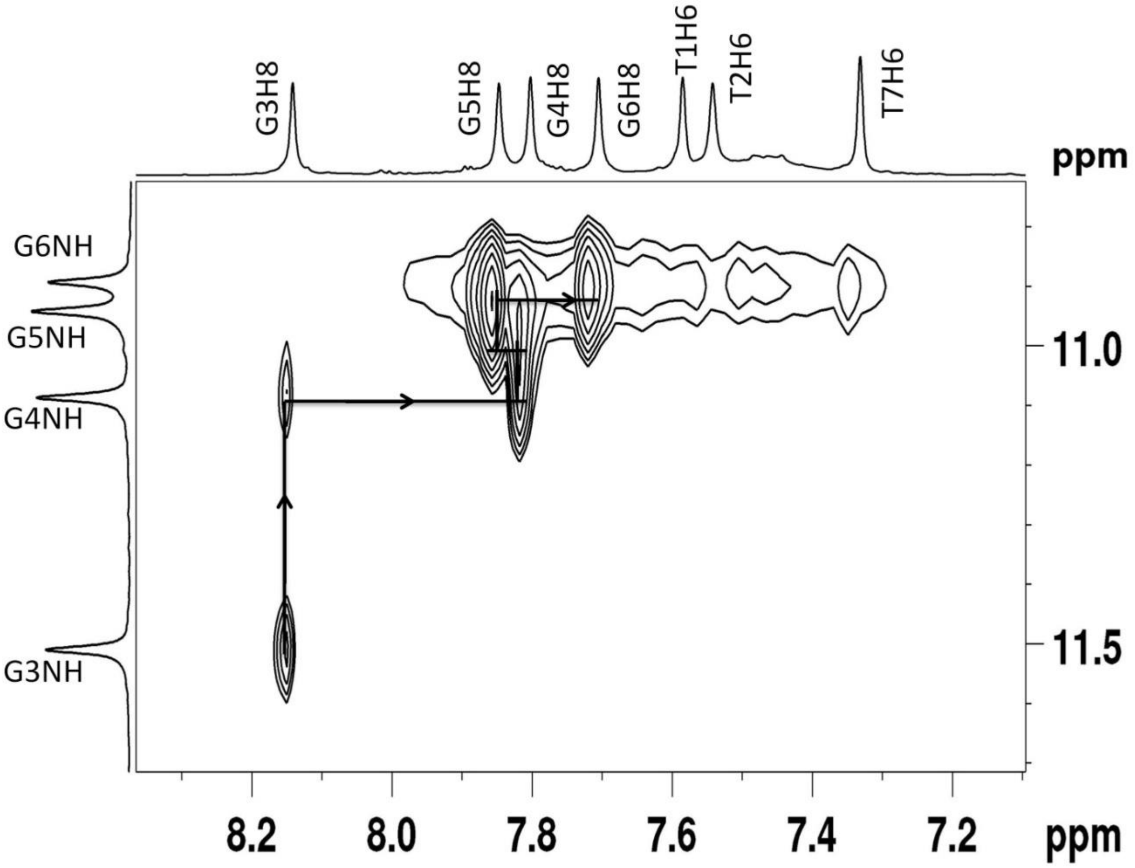
G3H8
G5H8
G4H8
G6H8
T1H6
T2H6
T7H6
ppm
G6NH
G5NH
G4NH
G3NH
11.0
11.5
8.2
8.0
7.8
7.6
7.4
7.2
ppm

e

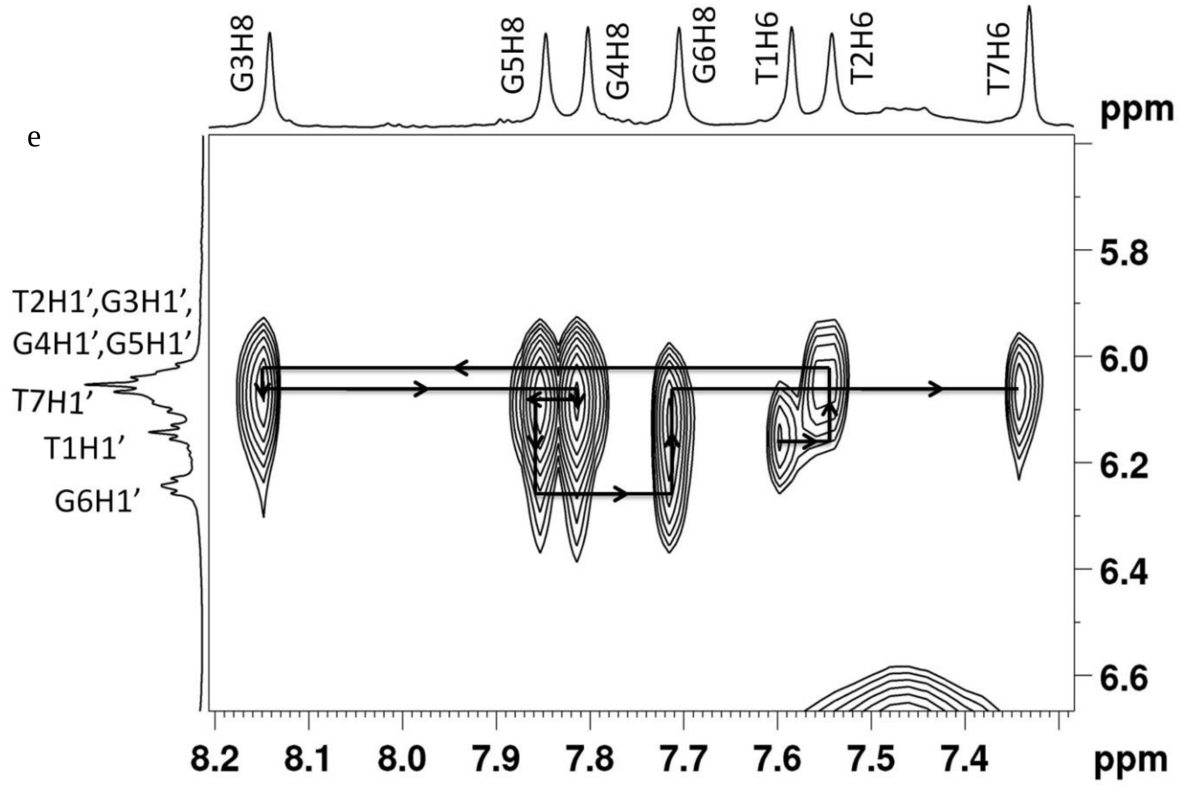

f

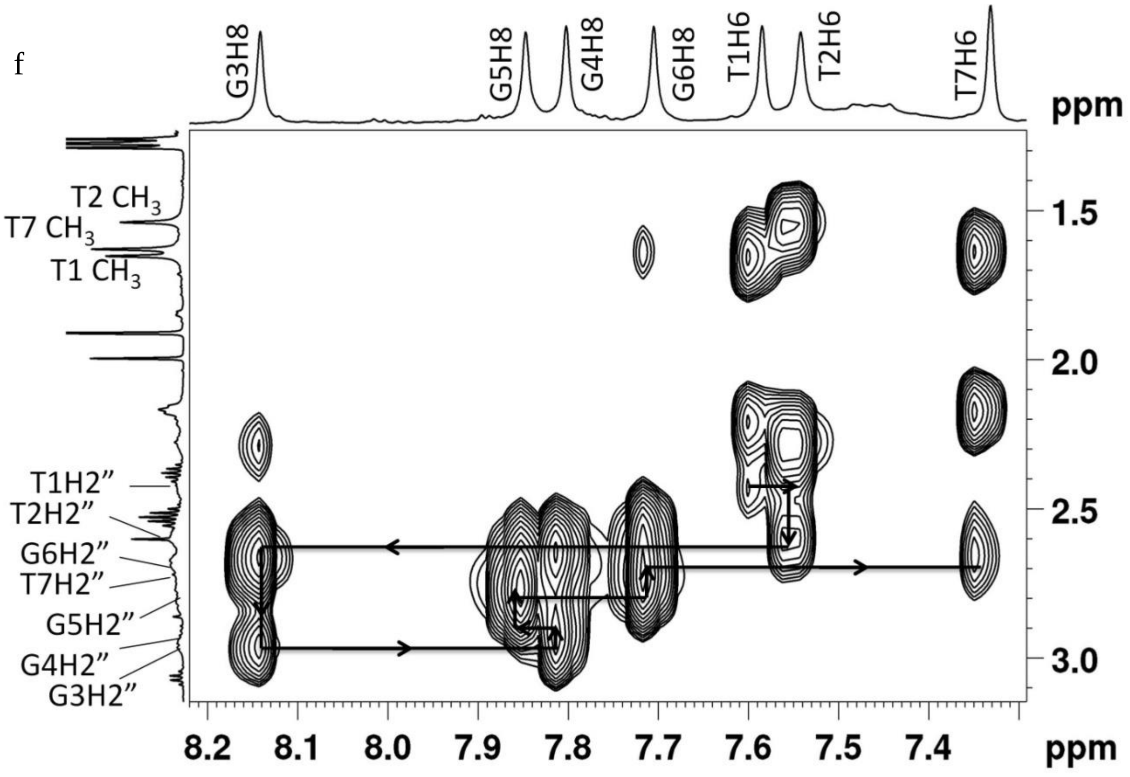

Figure S1 **a**,**b**) 1D ^{1}H NMR spectra of free [d-(TTGGGGT)]$_4$ in KBPES buffer containing 100 mM KCl (90% H_2O + 10% D_2O) at 25 °C. Expansion of ^{1}H-^{1}H 2D NOESY spectra of free [d-(TTGGGGT)]$_4$ showing sequential connectivity (black arrows) between c) imino protons d) adjacent imino and base protons e) base-sugar (H1') protons f) base-sugar (H2'/2") protons at τ_m = 250 ms in KBPES buffer (90% H_2O + 10% D_2O) at 25 °C.

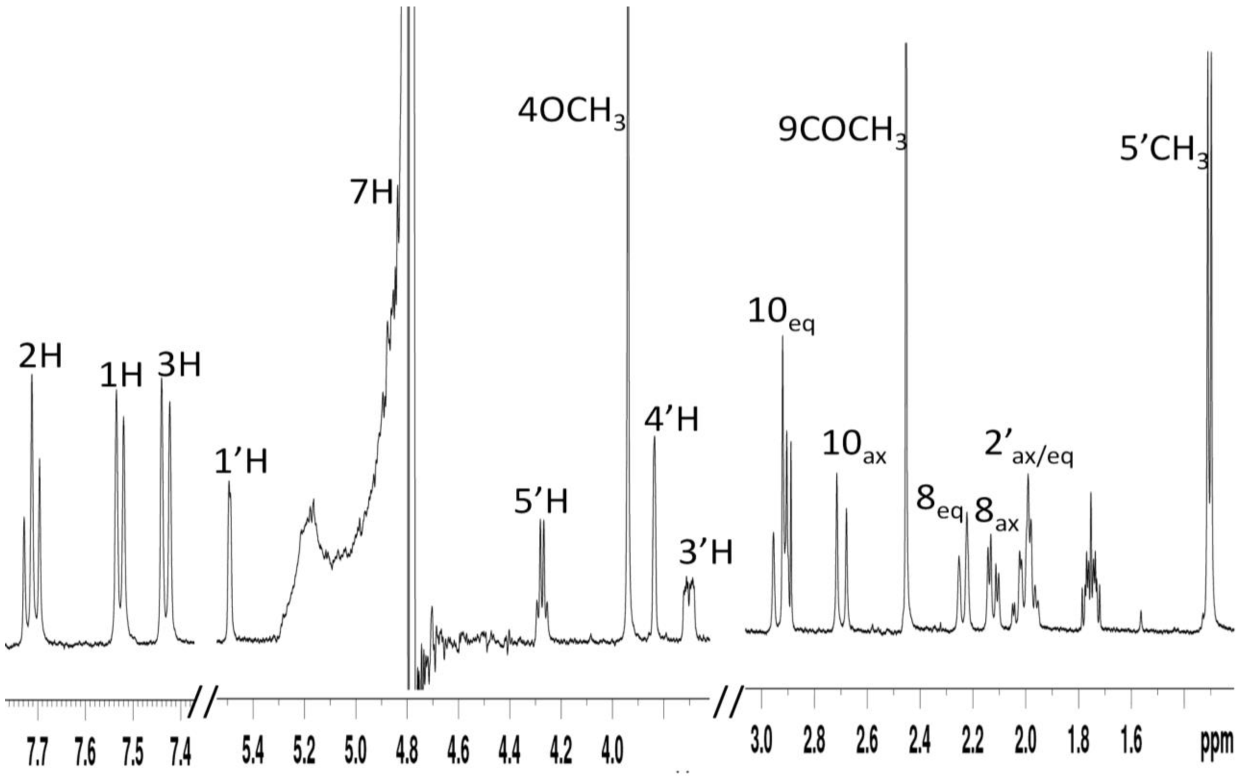

Figure S2: 1D ^{1}H NMR spectrum of free daunomycin in water (90% H_2O + 10% D_2O) at 25 °C.

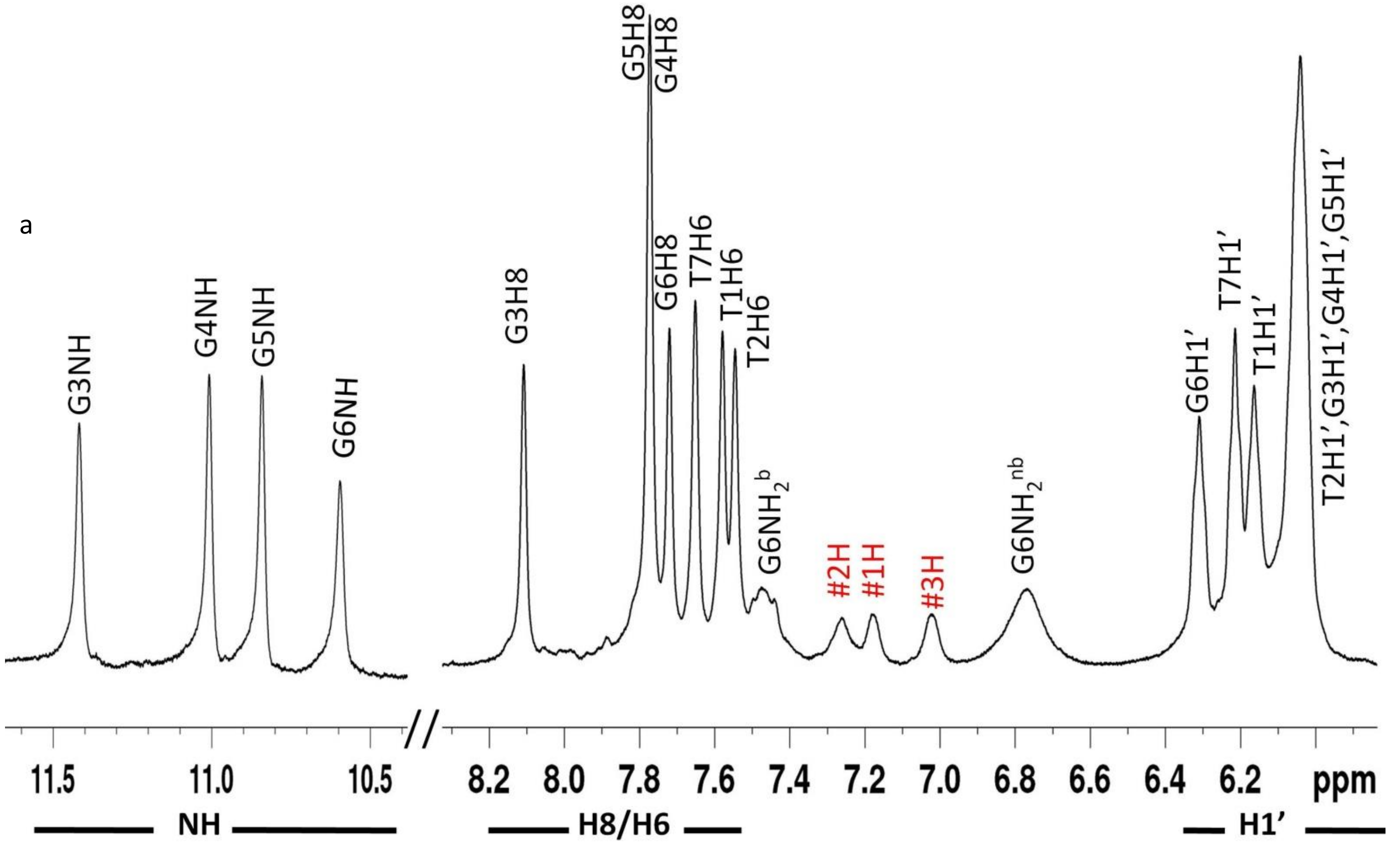

b

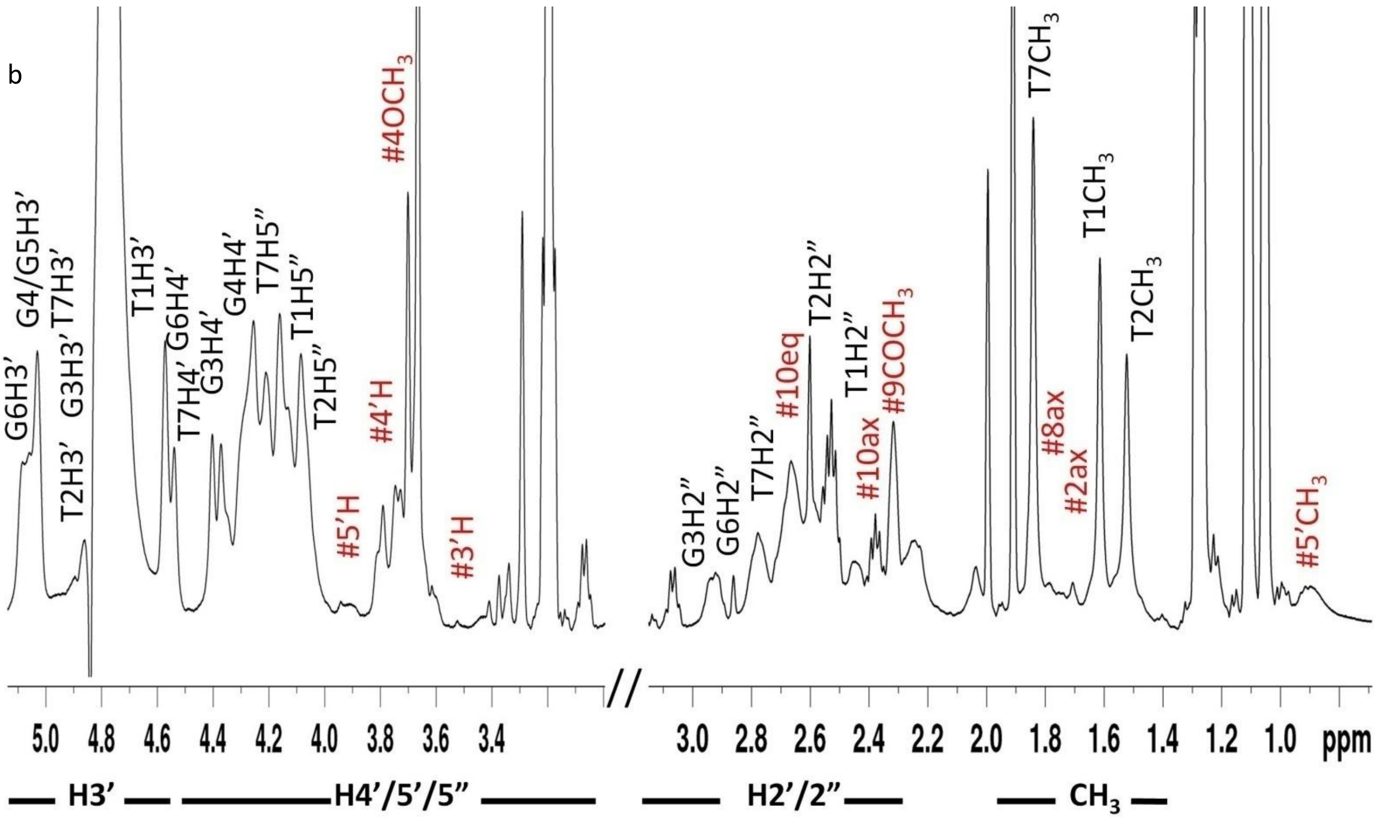
G6H3'
G4/G5H3'
T7H3'
G3H3'
T2H3'
T1H3'
G6H4'
T7H4'
G3H4'
G4H4'
T7H5"
T1H5"
T2H5"
#5'H
#4'H
#4OCH$_3$
#3'H
G3H2"
G6H2"
T7H2"
#10eq
T2H2"
T1H2"
#10ax
#9COCH$_3$
T7CH$_3$
#8ax
#2ax
T1CH$_3$
T2CH$_3$
#5'CH$_3$
5.0 4.8 4.6 4.4 4.2 4.0 3.8 3.6 3.4 3.0 2.8 2.6 2.4 2.2 2.0 1.8 1.6 1.4 1.2 1.0 ppm
H3'
H4'/5'/5"
H2'/2"
CH$_3$

c

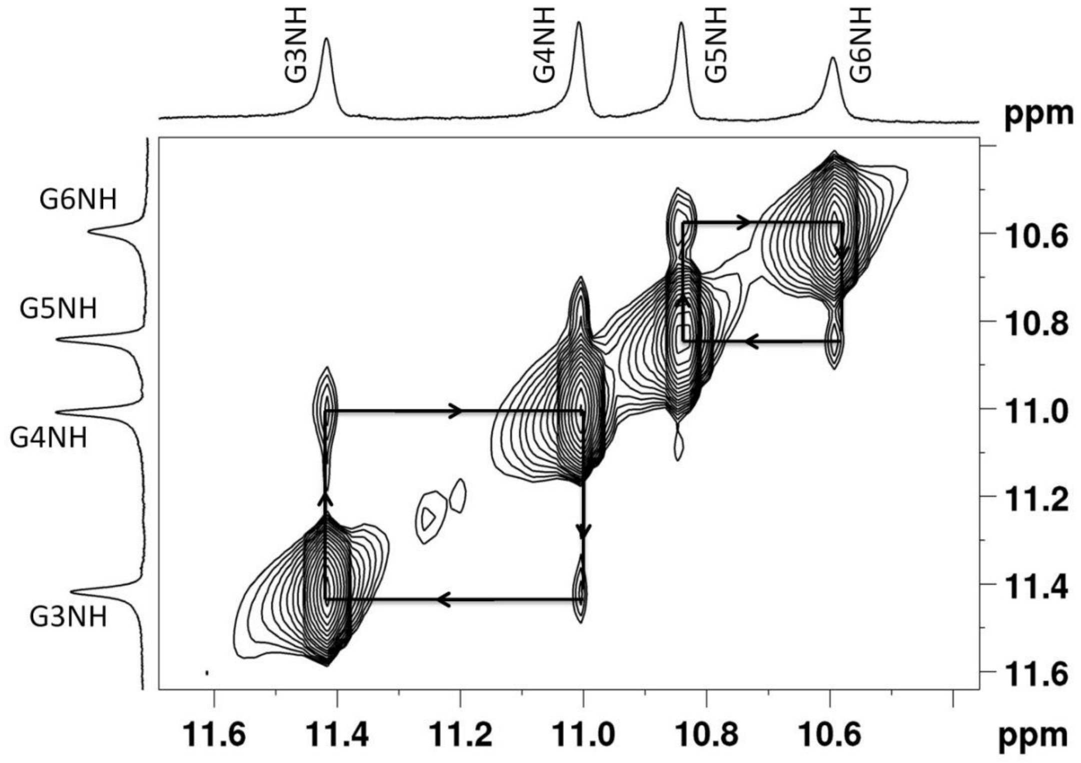
G3NH
G4NH
G5NH
G6NH
ppm
G6NH
G5NH
G4NH
G3NH
10.6
10.8
11.0
11.2
11.4
11.6
11.6 11.4 11.2 11.0 10.8 10.6
ppm

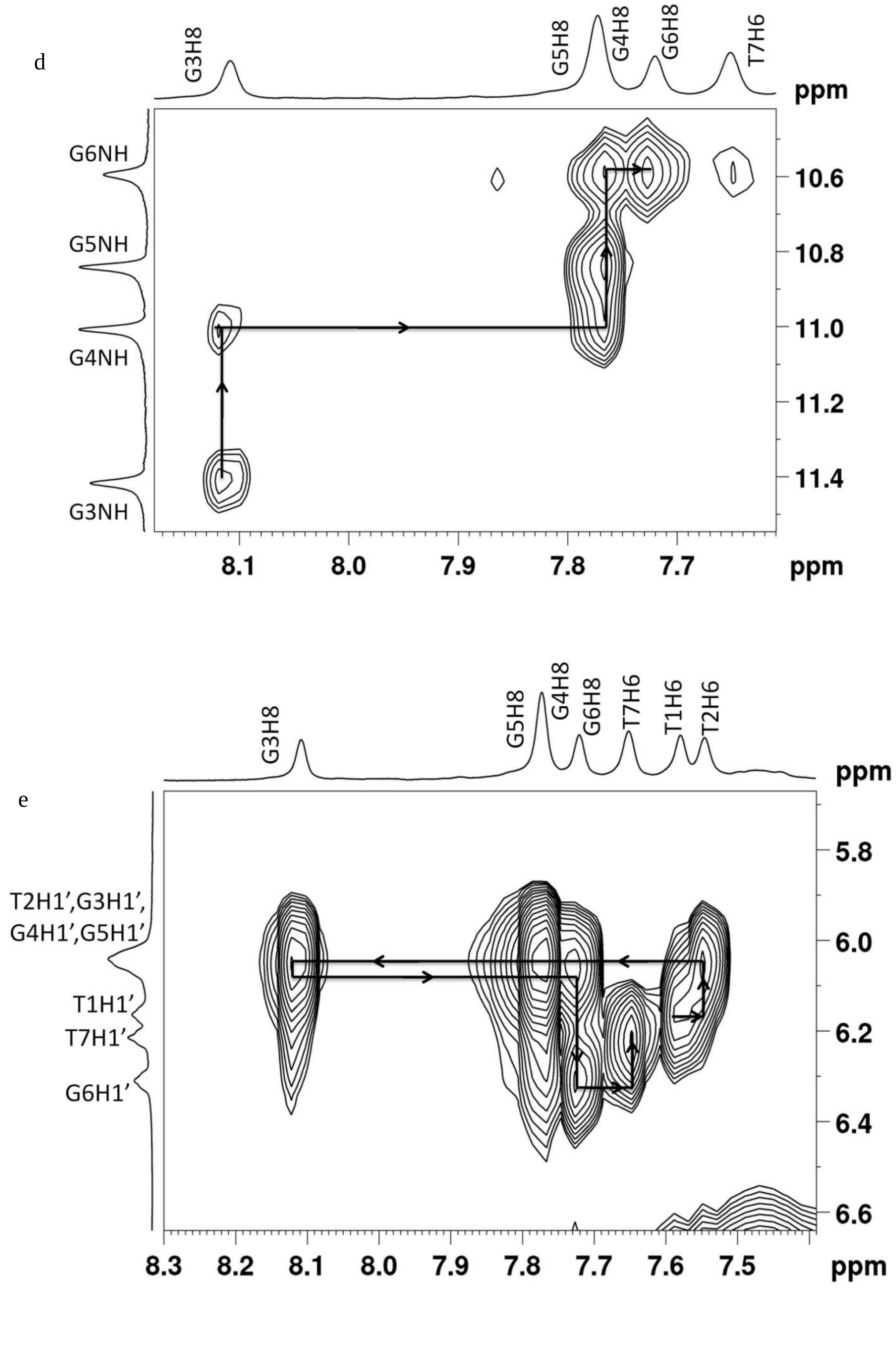

d
G3H8
G5H8
G4H8
G6H8
T7H6
ppm
G6NH
G5NH
G4NH
G3NH
10.6
10.8
11.0
11.2
11.4
8.1
8.0
7.9
7.8
7.7
ppm
e
G3H8
G5H8
G4H8
G6H8
T7H6
T1H6
T2H6
ppm
T2H1’,G3H1’,
G4H1’,G5H1’
T1H1’
T7H1’
G6H1’
5.8
6.0
6.2
6.4
6.6
8.3
8.2
8.1
8.0
7.9
7.8
7.7
7.6
7.5
ppm

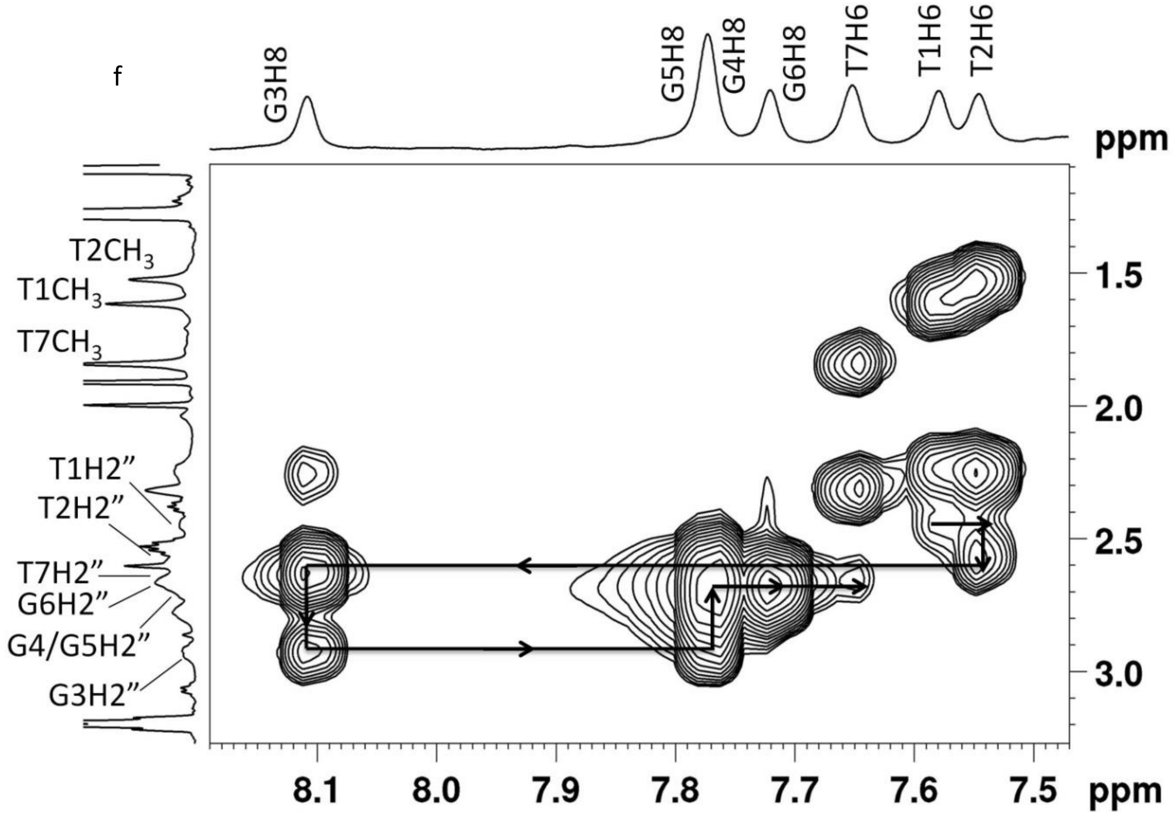

Figure S3 (a,b) 1D ^{1}H NMR spectrum of daunomycin-DNA [d-(TTGGGGT)]$_4$ complex at D/N = 2.0 in KBPES buffer containing 100 mM KCl (90% H_2O + 10% D_2O) at 25 °C. Daunomycin protons are represented by (#). Expansion of ^{1}H-^{1}H 2D NOESY spectra of daunomycin-DNA [d-(TTGGGGT)]$_4$ complex at D/N = 2.0 showing sequential connectivity (black arrows) between c) imino protons d) adjacent imino and base protons e) base-sugar (H1') protons f) base-sugar (H2'/2") protons at τ_m = 250 ms in KBPES buffer (90% H_2O+10% D_2O) at 25 °C.

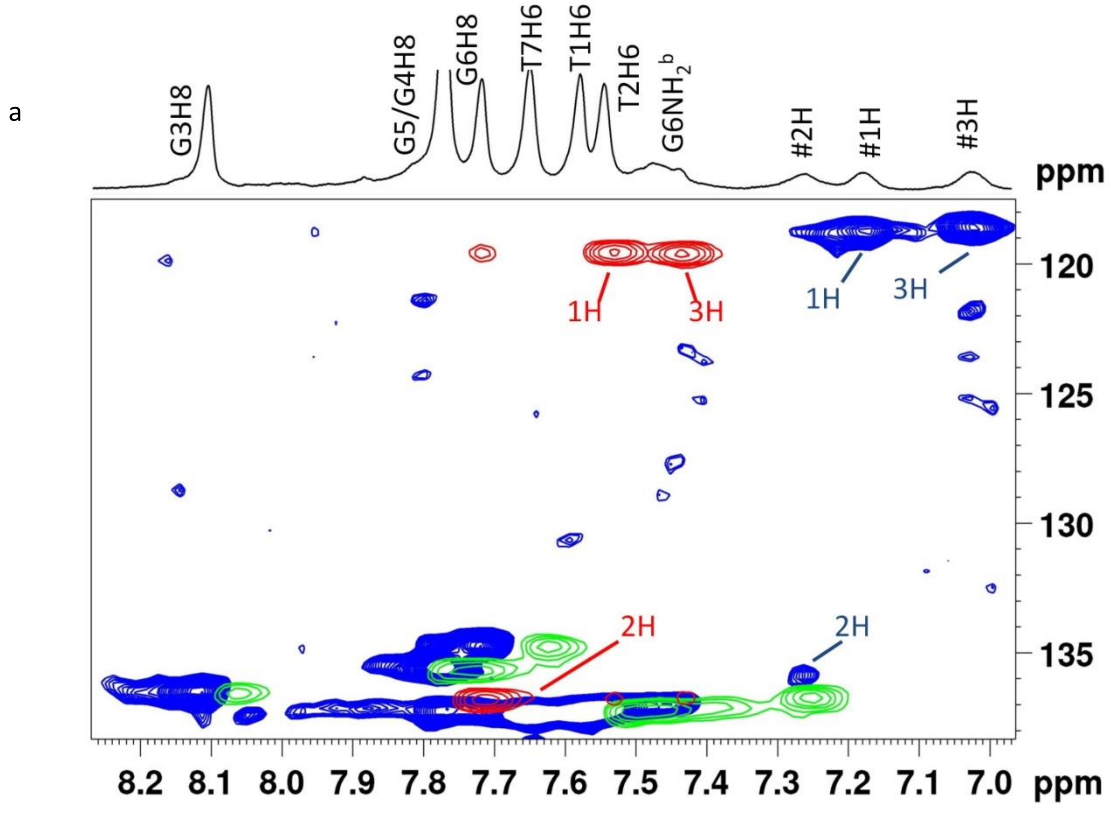

b

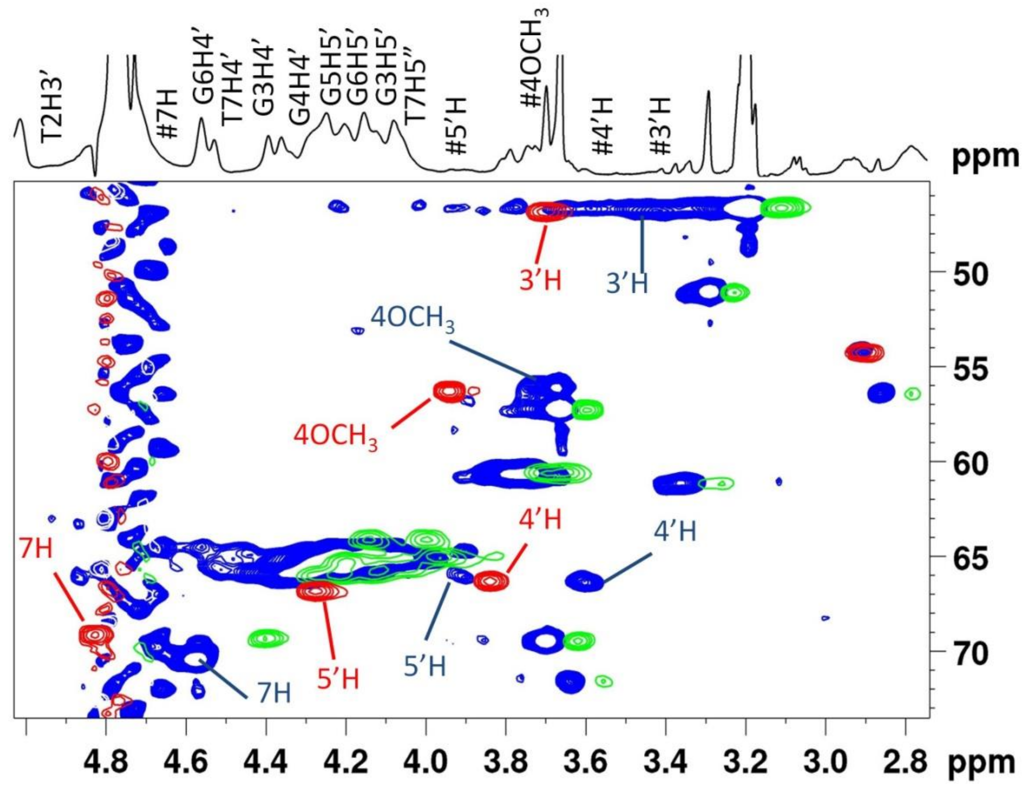

T2H3'
#7H
G6H4'
T7H4'
G3H4'
G4H4'
G5H5'
G6H5'
G3H5'
T7H5''
#5'H
#4OCH3
#4'H
#3'H
ppm
3'H
3'H
4OCH3
4OCH3
7H
4'H
4'H
5'H
5'H
7H
50
55
60
65
70
4.8
4.6
4.4
4.2
4.0
3.8
3.6
3.4
3.2
3.0
2.8
ppm

c

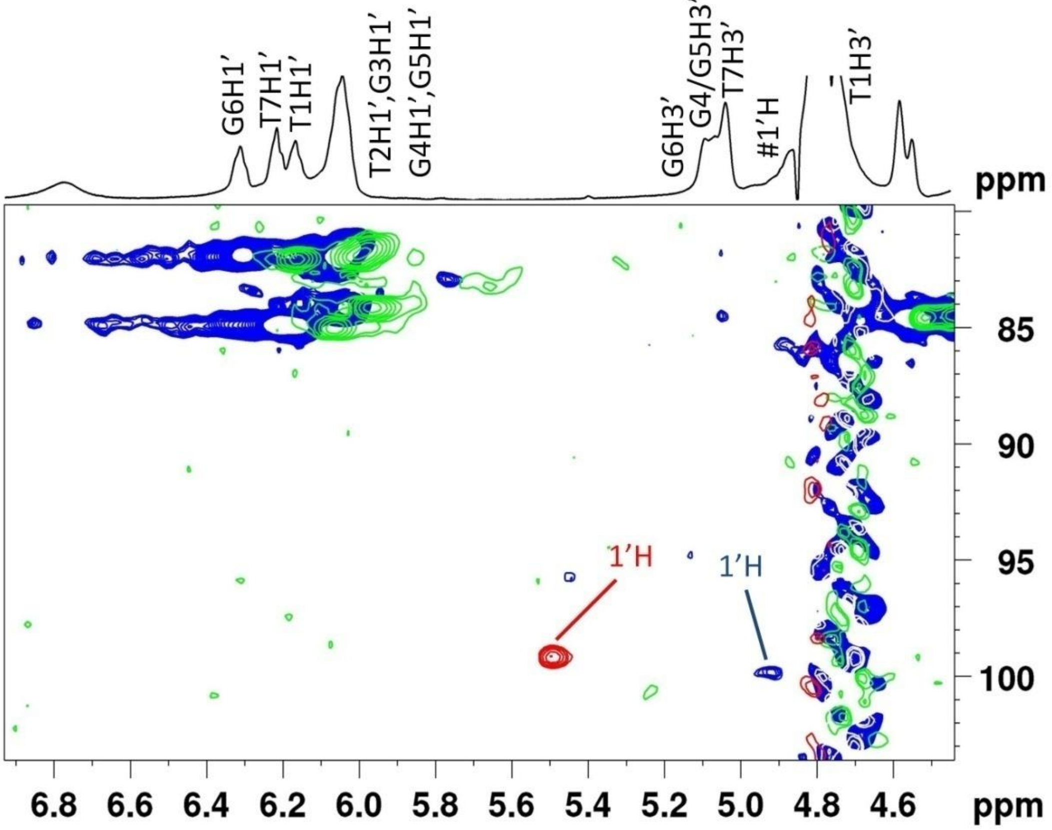

G6H1'
T7H1'
T1H1'
T2H1',G3H1'
G4H1',G5H1'
G6H3'
G4/G5H3'
T7H3'
#1'H
T1H3'
ppm
1'H
1'H
85
90
95
100
6.8
6.6
6.4
6.2
6.0
5.8
5.6
5.4
5.2
5.0
4.8
4.6
ppm

d

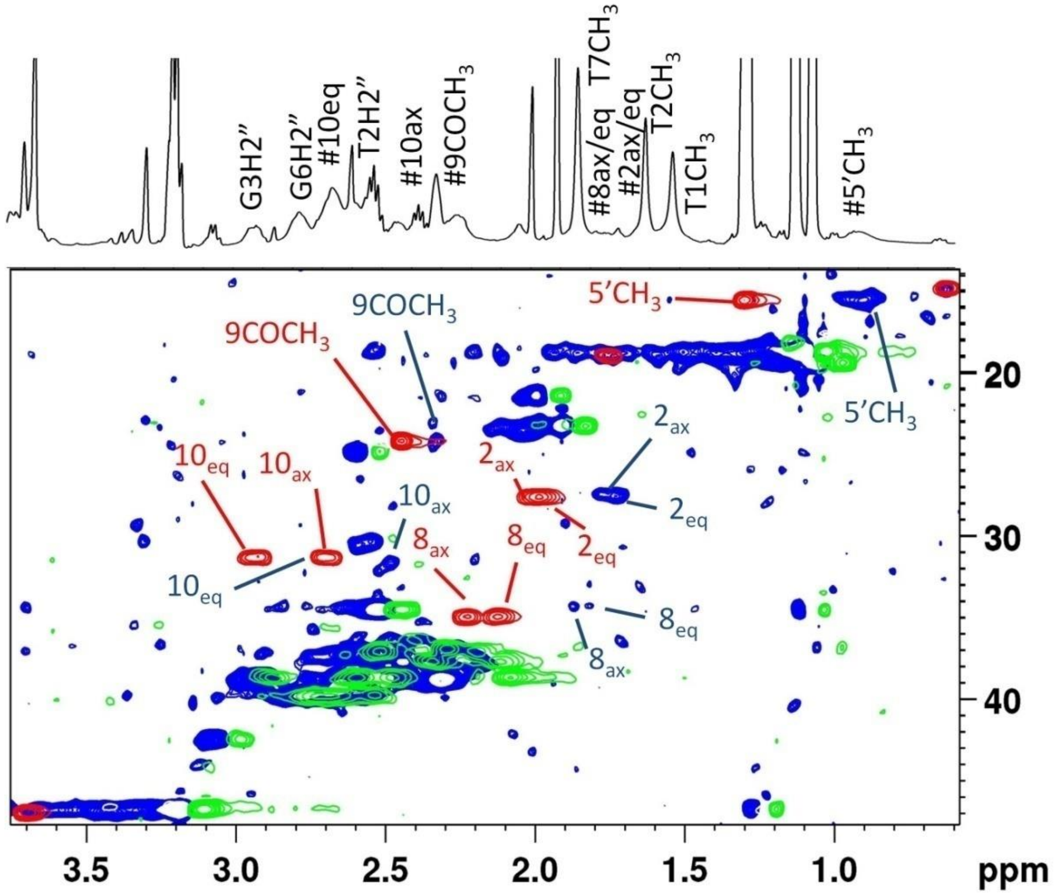

Figure S4 (a,d) Expansion of specific region of 2D ^{1}H-^{13}C HSQC spectrum showing overlay of free daunomcin (red), free [d-(TTGGGGT)]$_4$ (green) and daunomycin-[d-(TTGGGGT)]$_4$ complex at D/N = 2.0 (blue) in KBPES buffer (90% H_2O + 10% D_2O) at 25 °C. 1D ^{1}H NMR spectrum at the top of HSQC spectra is daunomycin-DNA [d-(TTGGGGT)]$_4$ complex at D/N = 2.0.

a b

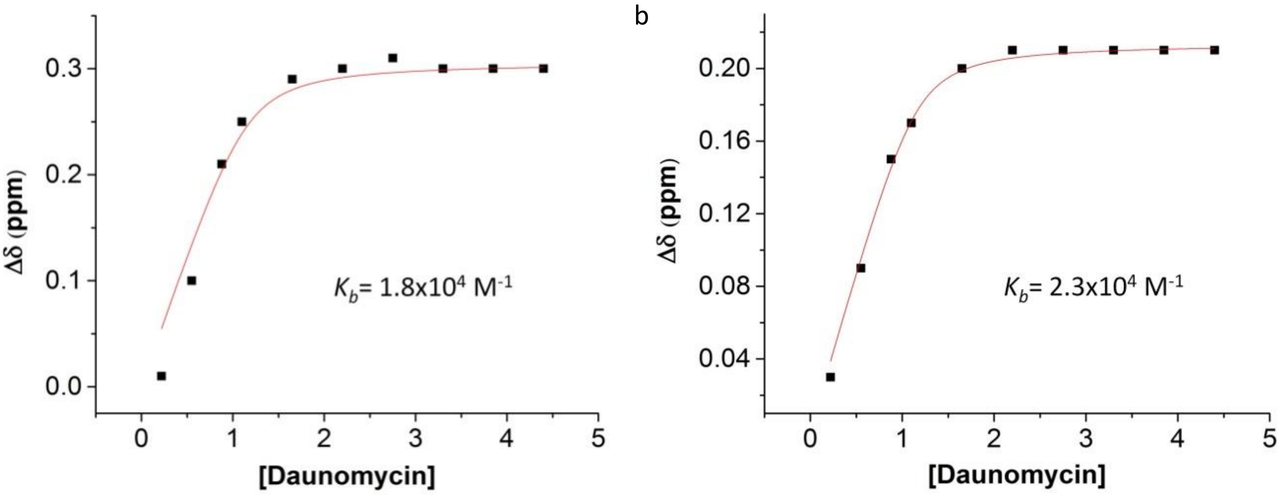

c d

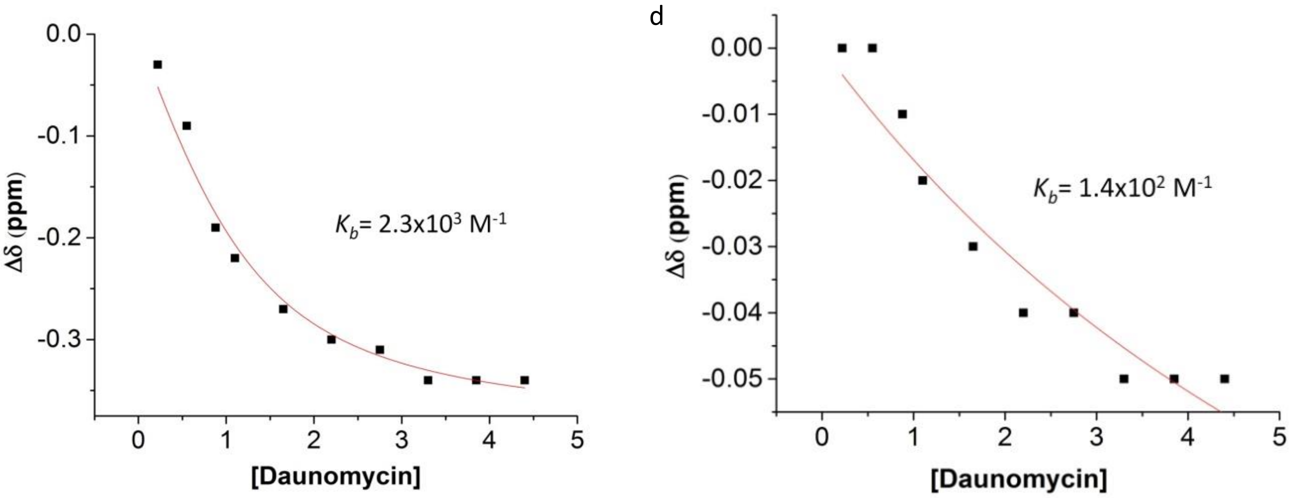

Figure S5 Nonlinear fitted curve (red) for simultaneous binding at two different sites in daunomycin-[d(TTGGGGT)]$_4$ complex of protons (a) T7H6, (b) T7CH$_3$, (c) G6NH and (d) T1CH$_3$. Plot shows change in chemical shift (Δδ) of protons at D/N = 0.2–4.0 as a function of daunomycin concentration (μM) showing binding constants (K_b) at 25 °C.

a

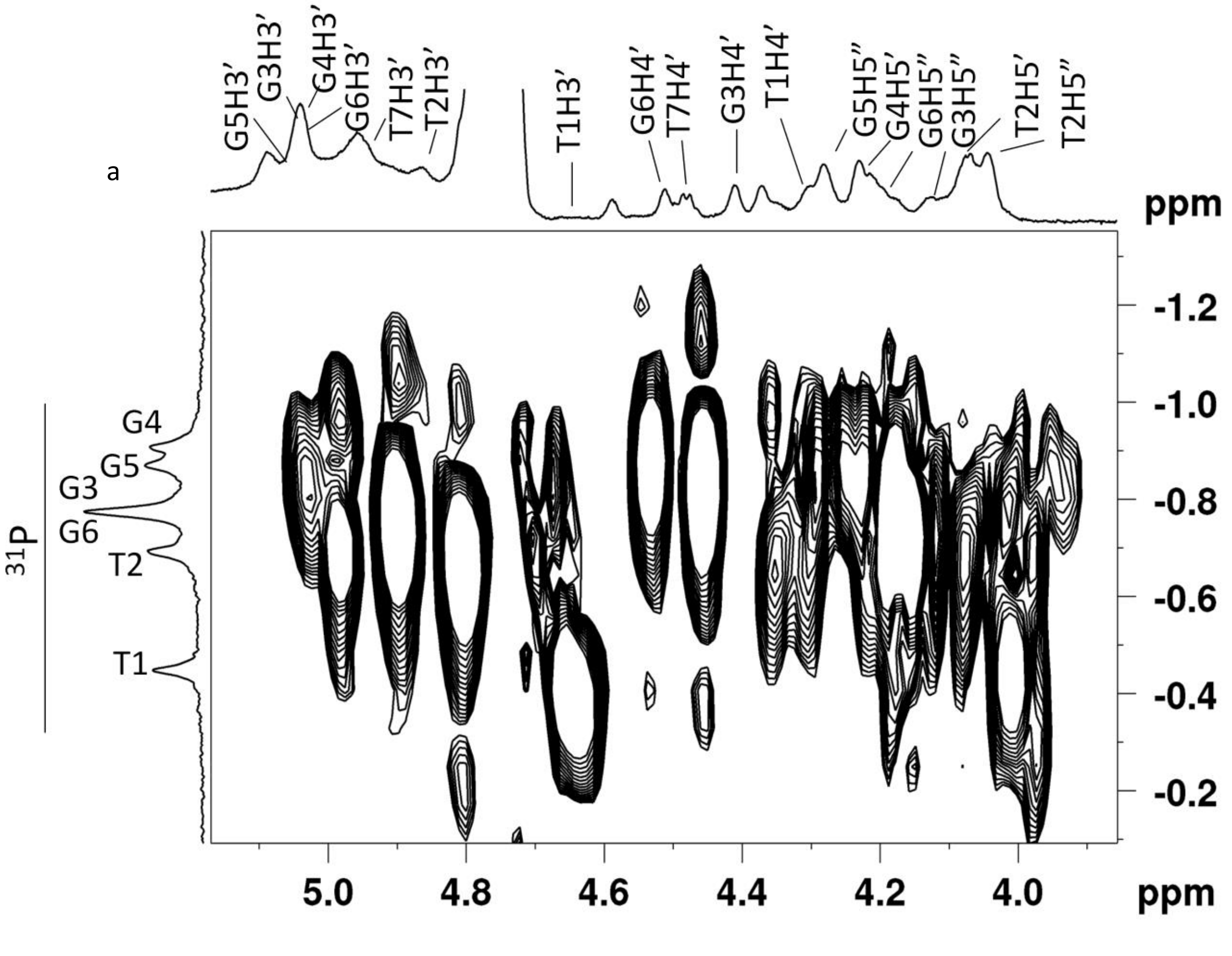

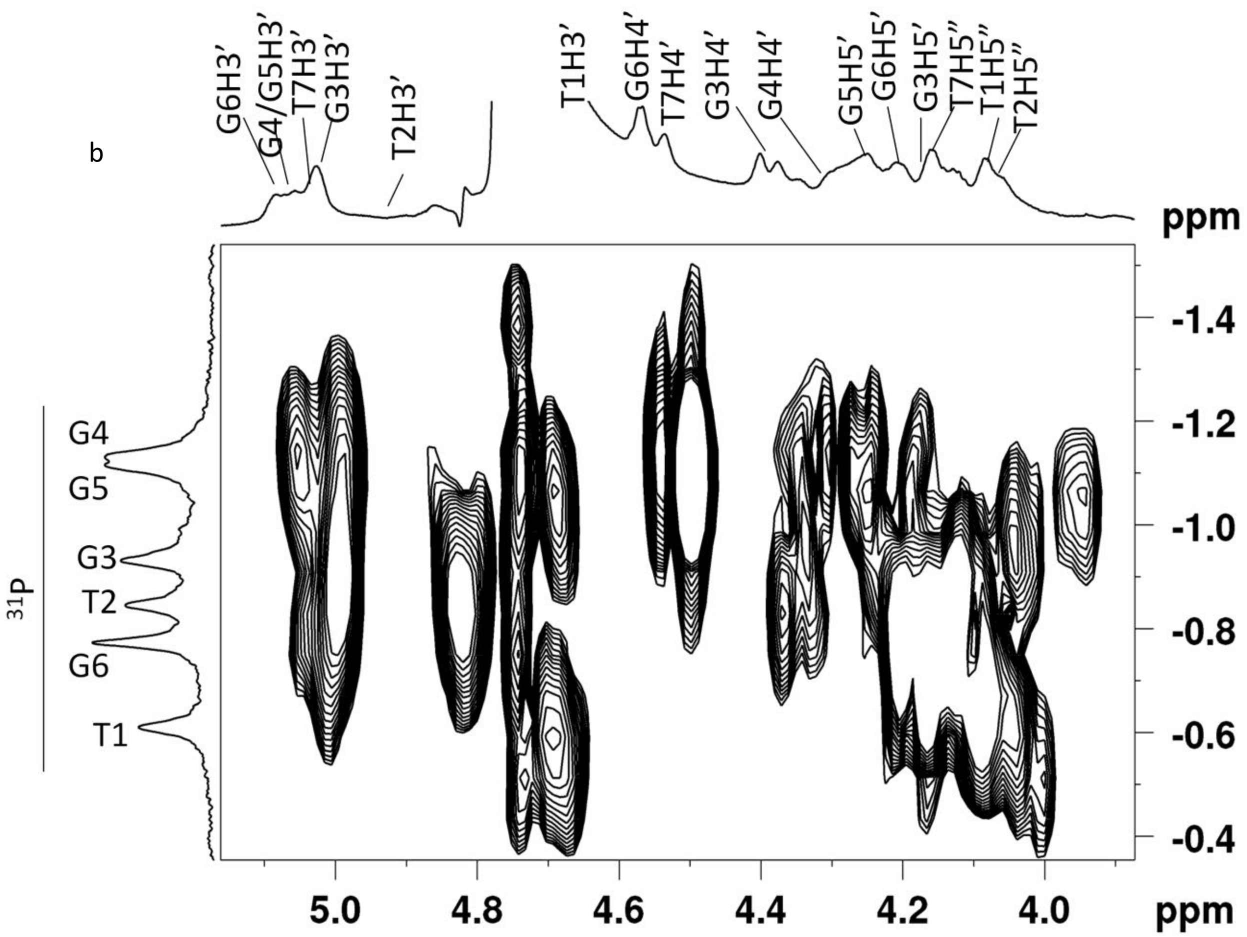

Figure S6: 2D ^{1}H-^{31}P HMBC spectra of a) free [d-(TTGGGGT)]$_4$ and b) daunomycin-[d-(TTGGGGT)]$_4$ complex at D/N = 2.0 complex in KBPES buffer (90% H_2O+10% D_2O) at 25 °C.

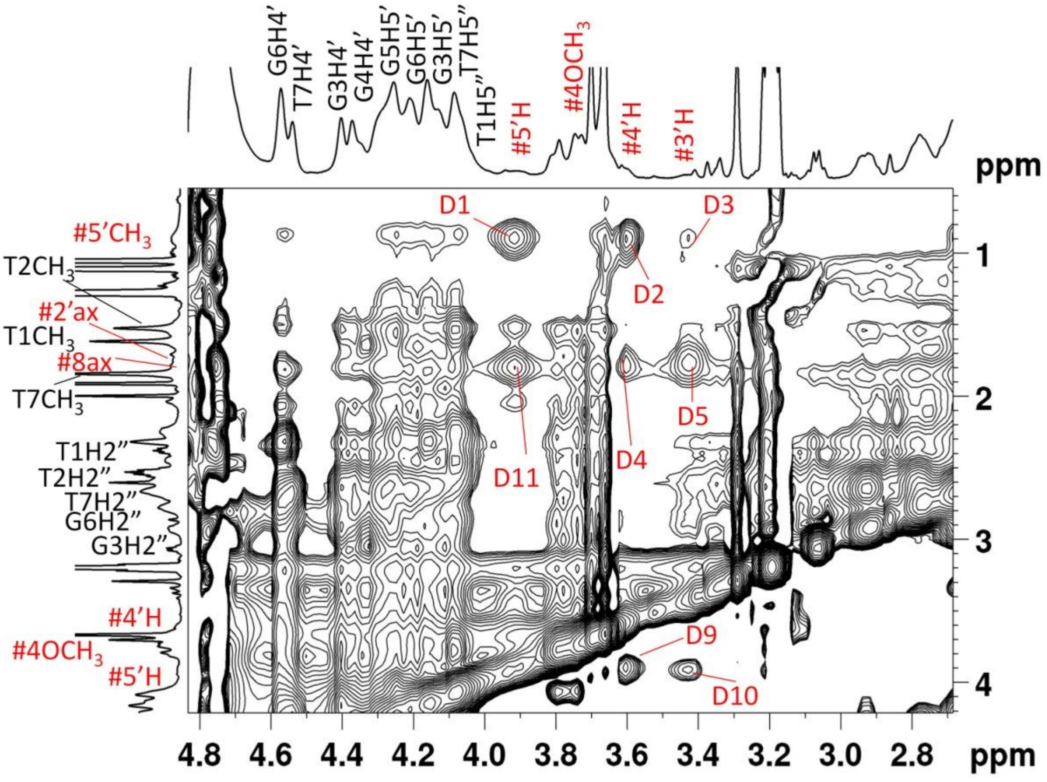

Figure S7: 2D ^1H-^1H NOESY spectra of daunomycin-[d-(TTGGGGT)]$_4$ complex at D/N = 2.0, mixing time (τ_m) = 250 ms at 25 °C. Expansion of specific region of NOESY spectra showing intra molecular NOE correlations between daunomycin protons 5'CH_3, 3'H, 4'H, 5'H, 2'$_{ax}$, and 8'$_{ax}$. Symbol # denotes daunomycin protons, D denote intra molecular daunomycin cross peaks (numbering of D done as per Table S3).

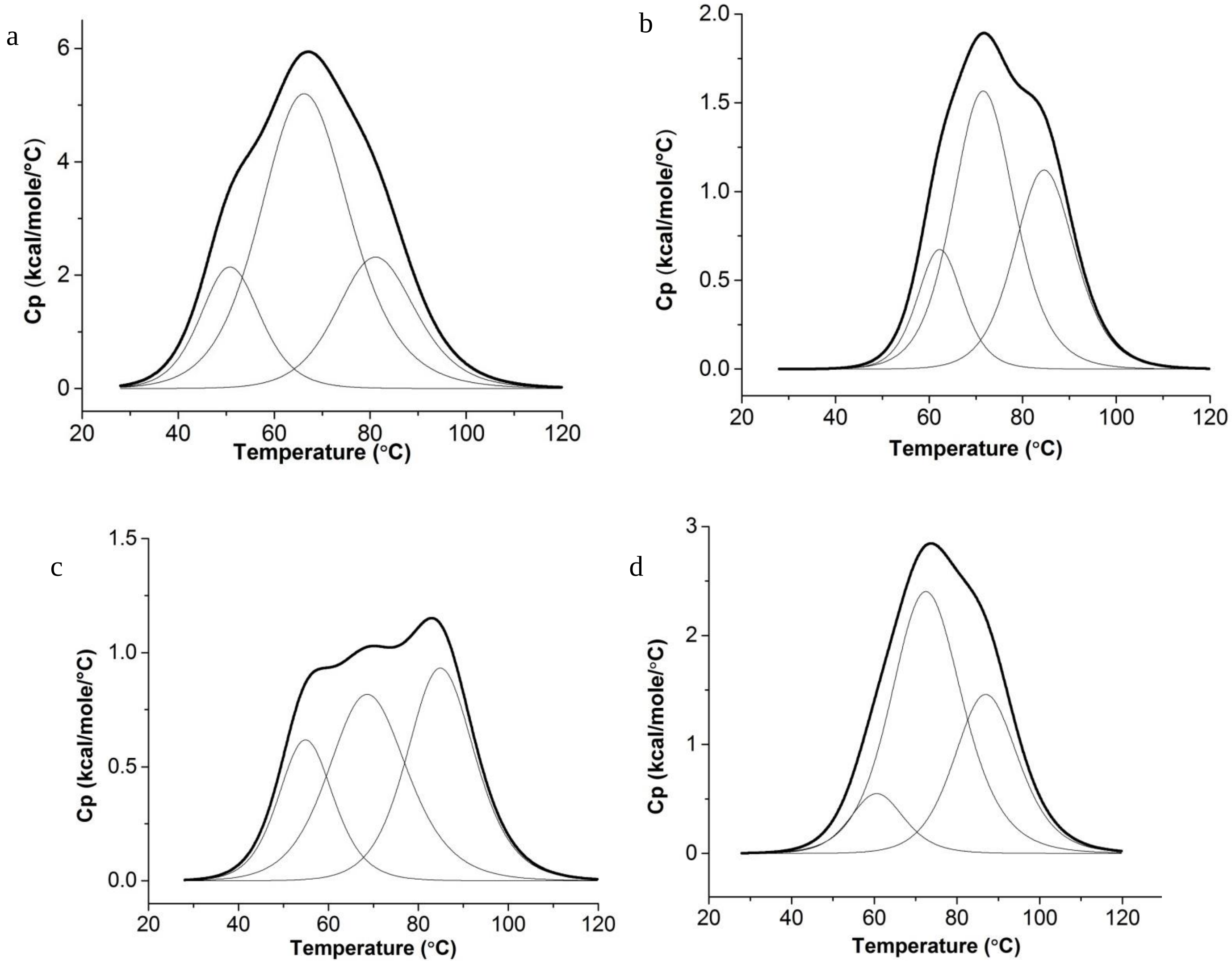

Figure S8: Differential Scanning Calorimetry (DSC) thermograms showing excess heat capacity as a function of temperature for daunomycin-[d-(TTGGGGT)]$_4$ complex at a-d) D/N = 0.5, 1.0, 3.0 and 4.0. All samples were prepared in phosphate buffer (KBPES) (pH 7.0) containing 100 mM KCl.
